# Oral microbiota, co-evolution, and implications for health and disease: The case of indigenous peoples

**DOI:** 10.1590/1678-4685-GMB-2023-0129

**Published:** 2024-01-22

**Authors:** Mariana Marcano-Ruiz, Thaynara Lima, Gustavo Medina Tavares, Maria Thereza Schmitt Mesquita, Luana da Silva Kaingang, Lavínia Schüler-Faccini, Maria Cátira Bortolini

**Affiliations:** ¹ Universidade Federal do Rio Grande do Sul, Departamento de Genética, Laboratório de Evolução Humana e Molecular, Programa de Pós-Graduação em Genética e Biologia Molecular, Porto Alegre, RS, Brazil.; 2 Universidade Federal do Rio Grande do Sul, Faculdade de Odontologia, Porto Alegre, RS, Brazil.; 3 Hospital de Clínicas de Porto Alegre, Instituto Nacional de Genética Médica Populacional, Serviço de Genética Médica, Porto Alegre, RS, Brazil.

**Keywords:** Oral microbiota, co-evolution, Native Americans

## Abstract

Evidence indicates that oral microbiota plays a crucial role in human health and disease. For instance, diseases with multifactorial etiology, such as periodontitis and caries, which cause a detrimental impact on human well-being and health, can be caused by alterations in the host-microbiota interactions, where non-pathogenic bacteria give way to pathogenic orange/red-complex bacterial species (a change from a eubiotic to dysbiotic state). In this scenario, where thousands of oral microorganisms, including fungi, archaea, and phage species, and their host are co-evolving, a set of phenomena, such as the arms race and Red or Black Queen dynamics, are expected to operate. We review concepts on the subject and revisit the nature of bacterial complexes linked to oral health and diseases, as well as the problem of the bacterial resistome in the face of the use of antibiotics and what is the impact of this on the evolutionary trajectory of the members of this symbiotic ecosystem. We constructed a *16SrRNA* tree to show that adaptive consortia of oral bacterial complexes do not necessarily rescue phylogenetic relationships. Finally, we remember that oral health is not exempt from health disparity trends in some populations, such as Native Americans, when compared with non-Indigenous people.

## The microbiota, coevolution, and implications for human health and disease

The microbiota is the community of microbes that colonize sites (or habitats), including within other living organisms. This means that symbiosis, a biological phenomenon where two or more species interact evolutionarily with different implications for each other, is necessarily present in the microbiota. 

The microbiota was largely ignored as a determinant factor of health and disease in living beings, partly due to methodological limitations that hindered the identification of its members and prevented a full understanding of their complex interactions. However, advances in large-scale sequencing techniques have produced a wealth of data that can now be analyzed using sophisticated bioinformatics tools, generating robust evidence that the microbiota plays a crucial role in the health and disease of living organisms. These methodologies have also been used to reveal how the members of this complex ecosystem evolve. 

The oral cavity of an animal contains diverse sites, including teeth, the gingival sulcus, gingiva, tongue, cheek, lip, and palate, as well as the dental biofilm (also known as plaque), and dental calculus (a form of hardened dental plaque) that forms at the teeth and the tooth-gum interface. In these sites, microorganisms inhabit and co-evolve with each other and with the host organism. This sensitive and complex ecosystem promotes a state of healthy balance (eubiosis) but also triggers disease in the host when an imbalance occurs, characterized by pathogenic microorganisms dominating over non-pathogenic ones (dysbiosis) (Kang et al., 2021; Radaic and Kapila, 2021).

For example, periodontal diseases (PDs), including gingivitis and periodontitis, have a multifactorial etiology, with genetic and environmental factors contributing to their development. PDs are characterized by dysbiosis in the oral microbiota, with chronic inflammation promoted by pathogenic microorganisms and their metabolic products leading to the destruction of the supporting tissues of the teeth. This destruction results in progressive loss of connective tissue attachment and bone resorption, significantly affecting chewing function and the appearance of affected individuals (Ferreira et al., 2017; Mohanty et al., 2019; Kang et al., 2021). 

Periodontal diseases represent a global public health concern, with a detrimental impact on human well-being and development. The PDs high incidence, prevalence, and life years lost/impacted, measured by disability-adjusted life years, have increased by ~8% from 1990 to the present, with particular impact in countries with low human development indices (Cui et al., 2022). 

The microbiota within the oral cavity is also a gateway to other colonizable sites in the body. Some oral microbiota species are considered risk factors for various systemic diseases, including certain types of cancer, cardiovascular, neurodegenerative, and metabolic diseases (Zhang et al., 2018; Dioguardi *et al.*, 2020). The relationship between the oral microbiota and diabetes is particularly interesting, as research has shown that periodontitis-associated bacteria can produce lipopolysaccharide, leading to insulin resistance, inflammation, and impaired blood sugar control. In addition, poorly controlled diabetes can contribute to oral disease, highlighting the two-way relationship between diabetes and periodontitis (Zhang *et al.*, 2018). Furthermore, microbes can metabolize a wide range of different medications and have the potential to alter their mechanism of action, indicating that drugs can influence microbiota composition, and vice versa (DeClercq et al., 2021).

In African bats, Lutz et al. (2022) found a link between dysbiosis in the oral microbiota and susceptibility to the Protozoan malarial parasites (*Plasmodium*spp). Specifically, the presence of *Pantoea agglomerans* bacteria can increase susceptibility to these parasites in bats. On the contrary, other authors showed that a protective microbiota suppresses parasite infection, reducing the need for host-based defenses. These results suggested that the microbiota can alter host-parasite co-evolutionary patterns and processes (Rafaluk-Mohr et al., 2022).

## Arms race, Red Queen, and Black Queen dynamics operating

It is important to note that natural selection can operate in various ways in a context where there are symbiotic relationships. For instance, natural selection can favor mutualism, a long-term codependency, when there is a benefit for each of the involved partners. However, mutualism requires that all members of the symbiosis maintain fitness, which makes them vulnerable over time to the evolution of “cheaters” or other forms of destabilization (Nelsen et al., 2020). Therefore, despite the fact that interactions involving mutualism can be stable for long periods, it can also be seen as an evolutionarily transitory condition since it can evolve into parasitism, where one organism, the parasite, lives at the expense of the other, the host. On the one hand, commensalism describes a symbiotic relationship in which one organism benefits without causing harm to the other species in the ecological relationship. Commensal bacteria and their products have been shown to play a crucial role in regulating the development, homeostasis, and function of innate and adaptive immune host cells, which makes it context indistinguishable from mutualism. Besides, under certain conditions, it may be challenging to distinguish between commensalism and parasitism because the protagonist member may exact a high cost that cannot be compensated by other members of the association (Leung and Poulin, 2008). The gradual emergence of microbial mutualists from parasitic ancestors is also well documented (Drew et al., 2021).

Moreover, a momentary loss of adaptive value in one of the species participating in the symbiotic relationship can trigger an evolutionary arms race or “Red Queen” dynamic (Van Valen, 1973). For example, in the case of antagonists, the pathogen and the host evolve to respond to the infection and defense strategies of each other, respectively, resulting in a situation where antagonistic organisms exert reciprocal selective pressure. This confrontation between antagonists will continue indefinitely unless one species goes extinct or when antagonists become evolutionary partners, ending the arms race or the Red Queen dynamic.

These two co-evolutionary types depend on whether there is reciprocal positive selection for host defenses and parasite infectivity (arms race) or balancing selection acting on the populations of the species in confrontation (Red Queen), which might be due to frequency-dependent selection or heterozygote advantage. Both models have been most often used to explain co-evolution between pairs of antagonists (*e.g.,* prey-predator, parasite-host interactions) (Rafaluk-Mohr et al., 2022), or when an escalatory co-evolution results in a shift from mutualism to parasitism. However, they are not the only ones. For instance, the microbiota members in caries and periodontal diseases can display a sophisticated structural and functional interdependence which could also be explained by the “Black Queen” hypothesis (Lamont et al., 2018). This hypothesis postulates, among other things, that pathogen agents that provide subversion of host immunity may discard expensive functions since they are maintained by other members of the bacterial community (Morris et al., 2012). In this case, one set of dominant pathogenic microorganisms does not necessarily eliminate the others; conversely, they recruit them as helpers. 

It should be noted, however, that these models/hypotheses are not mutually exclusive and do not claim to explain everything, particularly considering multispecific symbiosis, since the nature of the neutral, harmful, or beneficial interactions can change rapidly. They also do not include the role of the powerful purifying selection, drift, among other evolutionary processes.

In summary, the oral microbiota significantly influences human health and well-being and vice-versa. The interactions between microorganisms and the immune and inflammatory responses of the host are intricately correlated and can be affected by various other factors, including anthropogenic environmental conditions (*e.g*., oral hygiene, healthcare, use of antibiotics, dental visits, diet, and smoking) and genetic factors. As a result, diseases can develop, leading to associated clinical outcomes. Furthermore, poor oral health results in debilitating pain, limited social interactions, difficulties in eating and speaking, embarrassment, and a dramatic loss of quality of life. From an evolutionary perspective, the oral microbiota provides fascinating examples of the co-evolution of thousands of microorganisms and a particularly notable host, the primate *Homo sapiens*.

Other evolutionary dynamics operating in the microbiota are commented on in the Supplementary Material (Additional information regarding the main topics of the review).

## Oral microbiota, its composition, and its complexity

### How to identify bacterial taxonomic categories

The human oral cavity is home to approximately 1,000 different species, with bacteria being the most predominant (Zhang et al., 2018; Radaic and Kapila, 2021). Recent investigations have identified ultra-small bacteria belonging to the newly classified group known as “candidate phyla radiation” (CPR), as well as species belonging to the domains of life Archaea and Eukarya (Fungi and Protozoa), and viruses, including bacteriophages. 

Due to the prevalence and significance of bacteria in the human oral microbiota, this review provides a more detailed exploration of their diversity and role in health and disease contexts. Relevant information on other oral microorganisms, such as fungi and viruses, can be found in the Supplementary Material section.

Classifying life forms into a hierarchical system (taxonomy) and applying names to that hierarchy (nomenclature) is a challenging task. This challenge becomes particularly difficult in the case of microbes, such as bacteria. However, it is at a turning point due to modern research and bioinformatics methodologies.

According the “Human Oral Microbiota Database” (HOMD, 2023), the oral human bacterial community is dominated by 18 Phyla. Among these are Firmicutes, Bacteroidetes, Proteobacteria, Actinobacteria, Spirochaetes, and Fusobacteria, which account for 94% of the detected taxa but the species-level abundance for each oral site is variable.

HOMD indicates that there are 774 oral bacterial species, 58% of which are formally named and classified, 16% have not been classified/named yet, although cultivated. In contrast, 26% are known only as non-cultivated phylotypes, *i.e*., an active group of individuals that are phylogenetically similar but have not been reproduced in culture yet. 

The taxonomy adopted by HOMD provides a tentative naming scheme for currently unnamed taxa based on the *16SrRNA* sequence phylogeny so that strain, clone, and probe data from any laboratory can be directly linked to a reference scheme with a stable nomenclature. The 16SrRNA molecule, is required for the initiation of bacterial protein synthesis and the stabilization of correct codon-anticodon pairing in the 30S ribosomal subunit during mRNA translation (Wimberly et al., 2000).

For decades, hypervariable regions of *16SrRNA* have been used to identify microorganisms, particularly bacterial and archaeal species, due to their evolutionary rate and other characteristics that can provide specific phylogenetic signatures. However, it is important to note that the classification of microbes based on *16SrRNA* sequence relationships has limitations, particularly in accurately representing phylogeny at different taxonomic levels. Nevertheless, culture-independent techniques are now producing thousands of metagenome-assembled genomes. In addition, metagenome-based identification facilitates the assessment of functional dynamics within the microbial community.

By combining vertically inherited single-copy protein genes, which are increasingly available from metagenome data sets, excellent resolution can be achieved, resulting in a more comprehensive representation of microbial diversity. Although concatenated protein trees have their limitations due to some level of lateral gene transfer, varying rates of evolution, and recombination, they have been used and considered the best approach for a reference bacterial phylogeny (Parks et al., 2018; GTDB taxonomy is publicly available at the Genome Taxonomy Database (2023)). Other initiatives can be found in “The Human Microbiome Project Consortium” (2023). The Supplementary Material in this review also explores other aspects of the methodologies used to classify microorganisms.

### The bacterial complexes

Although there is individual variation, most unrelated healthy people are believed to share a core oral microbiome (Caselli et al., 2020), the collective genome of the oral microbiota. The co-evolution of this “healthy” core microbiome and the host genome, and their corresponding adaptive phenotypes, promotes an evolutionary state of apparent equilibrium, another way to define eubiosis. As already mentioned, this momentary stability can be disrupted when a group of bacteria triggers microbial imbalance (dysbiosis), provoking host diseases. These pathogenic microorganisms can evade the host immune response, surviving and reproducing in an oxidative stress-rich environment, such as the periodontal pocket, or thrive under an acidic environment found on supragingival biofilm associated with carious lesion development. In this scenario, a set of microorganisms cooperates in a co-evolutionary relationship. However, they harm the host, triggering a new cycle of arms race or Red Queen dynamics, considering the host and the pathogen, which can be extended to other members of the microbiota that pose as antagonists. The Black Queen hypothesis can also be evoked between the microbes to explain at least parts of these findings since, in many cases, there is no complete overlap among the species.

The observation that there is a succession of bacterial prevalence from a healthy oral state to a diseased oral state led Socransky et al. (1998) to utilize various available techniques to classify subgingival biofilm bacteria into color groups, as shown in Table S1. 

The “red complex” is an adaptive consortium of pathogenic bacteria species present in the human oral microbiota that are strongly linked to oral diseases. These bacterial species include *Tannerella forsythia, Treponema denticola*, and *Porphyromonas gingivalis*, and their concomitant appearance is mainly due to their synergistic interaction (Socransky et al., 1998). Recent studies have shown that these bacteria exhibit metabolic interdependence (Nayak et al., 2018), signaling a scenario of obligatory mutualism between them. 

Bacteria such as *Campylobacter rectus, Fusobacterium nucleatum,* and *Eubacterium nodatum,* belong to the “orange complex” and are considered pathogenic agents (Table S1). They also serve as a bridge between early colonizers and the red complex bacteria. The oral colonization sites and the number of the red complex bacteria increase with an increase in colonization by the orange complex (Mohanty et al., 2019; Kang et al., 2021). Subjects who did not respond adequately to antibiotic treatment (“refractory” patients) may have 80% of their bacterial microbiota composed of red and orange species (Socransky and Haffajee, 2005).

Dental caries is another multifactorial oral disease, and it is considered the most prevalent human disease. Dental caries is caused by an overgrowth of acid-tolerant bacteria in the oral cavity, such as *Streptococcus mutans,* but the presence of red complex bacteria has been found to be positively associated with an increased risk of this pathology in different populations (Ozga et al., 2016; Inquimbert et al., 2019). 

Species of the genus *Streptococcus* make up the “Yellow complex”, while the “Green complex” is composed of species of the genera *Campylobacter, Capnocytophaga, Aggregatibacter*, or *Eikenella*. The “purple complex” comprises *Veillonella parvula* and *Actinomyces odontolyticus* (*Schaalia odontolytica*), while the species of genus *Actinomyces* are recognized members of the “blue complex” (Socransky and Haffajee, 2005) (Table S1). These last four conglomerates are early colonizers of the tooth surface and firmly related to periodontal health.

The presence of certain species of oral microbiota, such as *P. gingivalis* (red) and *Prevotella intermedia* (orange), has also been associated with an increased risk of cancer (Pignatelli et al., 2022), while some studies show that the nature of the oral microbiota can vary considering diabetic and non-diabetic subjects, although the specific differences are not clear (Zhang et al., 2018). However, some studies reveal that subjects with good glycemic control have a lower detection of*Filifactor alocis*as compared to fair- and poor-glycemic-control subjects. The co-occurrence of this microorganism with the red *T. forsythia* in diabetic subjects with chronic periodontitis indicates a synergistic collaboration between them (Pandian et al., 2023).

Additionally, studies have linked PDs and *P. gingivalis* to Alzheimer disease. This red complex bacterial species was detected in the brains of Alzheimer patients and have shown to increase amyloid plaque production in mice (Abbayya et al., 2015; Teixeira et al., 2017; Dioguardi *et al.*, 2020; Radaic and Kapila, 2021). The inflammatory process associated with PDs may also intensify inflammation in the central nervous system, contributing to the occurrence of Alzheimer disease (Dioguardi *et al.*, 2020).

In addition to the oral bacteria mentioned above, some opportunistic pathogens found in the oral cavity and other body sites can also significantly threaten overall health. For instance, *Staphylococcus aureus*, which is often an asymptomatic colonizer, can turn into a virulent and multiresistant pathogen that causes infection in the oral cavity and can spread throughout the body, making it of great clinical significance (Howden et al., 2023). Noteworthy, *S. aureus* isolates prevented the growth of *T. denticola* and *P. gingivalis,* suggesting a certain level of antagonism with these red complex bacteria (Suzuki et al., 2013). 

Interestingly, the studies show that the microbial community in the context of poor oral health is enriched with species traditionally classified as red-complex bacteria. However, they do not entirely replace the non-pathogenic ones. In other words, the virulence and pathogenicity of the red/orange species against the host are thought to be enhanced not only for their synergistic interactions with each other but also with other members of the oral microbiota. This finding indicates that the Black Queen hypothesis can be part of the phenomena that explain the co-evolution of these oral microorganisms. 

## Diversity of the oral microbiota in humans and other primates

Some studies with non-human primates, extinct hominins (*e.g., Homo neanderthalensis*), and ancient *Homo sapiens* populations have been carried out, introducing temporal depth and a macroevolutionary perspective regarding the nature of the oral microbiota. 

For example, chimpanzees (*Pan troglodytes*) and bonobos (*Pan paniscus*) that inhabit different African sanctuaries had a salivary microbiota that was more similar to each other compared to the humans (employees of each sanctuary). Furthermore, the two human groups compared also showed similarities in their microbiota, a result consistent with the phylogenetic relationships and physiology of the hosts (Li et al., 2013). 

Other studies also revealed that the dental calculus microbiota of chimpanzees has a higher frequency of bacteria of phyla Bacteroidetes and Fusobacteria, while humans have higher proportions of Firmicutes and Proteobacteria species (35% and 19%, respectively, of the species already identified in the oral cavity), but the causes of these differences remain unknown (Ozga et al., 2019; HOMD). Ozga *et al.* (2019) did not find a significant association between one particular bacterial genus and the presence of caries or the absence of teeth in chimpanzee. Besides, PDs have been documented in captive and wild great apes, but the connection between these and the red complex bacteria in the oral cavity of *Pan* spp. is not known yet (Ozga *et al.*, 2019). Another example can be found in the Supplementary Material section.

Analysis of dental calculus from five Neanderthals revealed that meat consumption contributed to substantial variation in their oral bacterial community (Weyrich et al., 2017). Weyrich (2021) used modern farmers and hunter-gatherers as proxies to understand how changes in diet have been affecting the oral microbiota of *Homo sapiens* over time. The author suggests that in Europe, the evolution of the oral microbiota has been shaped by interactions with Neanderthals in ancient times as well as adaptation to agriculture and industrialization in more recent times.

## Genetic data in the context of the human oral microbiota

Within an ecosystem where powerful evolutionary forces are at play, signatures in the microbiome and host genome are expected to occur. Below are some illustrative examples.

### Resistome as an example of the bacterial response

Antibiotics are vital for treating infectious diseases, including oral pathologies. However, antibiotics act as agents of natural selection. Their excessive and inappropriate use has led to the rapid development and spread of microbial resistance, which is a serious global public health challenge. If nothing is done, the number of human annual deaths attributable to antimicrobial resistance will rise from the current ~700,000 to ~10 million in the year 2050 (O’Neill, 2016).

Therefore, identifying the “resistome” of the microbiota, which refers to the set of functional genes responsible for antibiotic resistance (ARGs) present in bacteria, become critical due to accelerated emergence of antibiotic-resistant oral pathogens. In addition to antibiotics, metal and metal oxide nanoparticles have been studied due to their antibacterial properties in dentistry. Consequently, genes responsible for metal resistance (MRGs) are investigated due to the decay of their antibacterial activity over time (Kang et al., 2021). The co-occurrence of ARGs and MRGs signals the existence of multiple selective pressures. Kang *et al.* (2021) conducted a metagenomics study, considering dental plaque samples from healthy and periodontitis subjects and those after successful treatment. Genes that confer resistance to Tetracycline [such as *tet(32)* and *tetW*] and multiple drugs (such as *emrA, emrB,* and *mdtG*) were highlighted by the authors, but the number of ARGs in patients was significantly higher than in the group of healthy volunteers. Furthermore, they found a significant change in the profiles of ARGs and MRGs of the microbial community present in dental plaque due to treatment for periodontitis (Kang *et al.*, 2021). 

ARGs and MRGs are often transferred between bacteria via mobile genetic elements, such as conjugative plasmids and transposons, promoting bacterial genome mutability and evolution. For example, transposons belonging to the Tn916 family can transfer resistance to various pathogens, including commensal and pathogenic oral bacteria (Kang et al., 2021). 

Sukumar et al. (2023) investigated the oral resistome and its role in dental caries in 221 twin children at three different time points T1 (6.7 ± 2.7 months, absence of teeth), T2 (1.6 ± 0.4 years, primary/deciduous/baby teeth only), and for T3 (8.5 ± 1.2 years, mixed dentition). They found the co-occurrence of Tn916 transposase and ARGs in 50%, 32%, and 35% of individuals at T1, T2, and T3, respectively. This co-occurrence was predominantly observed in *Streptococcus* species across all time points (78%). Moreover, the study showed that over time, Tn916 was detected in a greater number of species and in combination with more ARGs. For instance, at T1, the co-occurrence of Tn916 with *tet(M)* and *ermB* was found in *Streptococcus oralis*, but by T3, the same species was associated with three additional ARGs: *mefA, msrD*, and *lsa(C)*. The investigation revealed a decrease in both ARGs and species abundance in dental caries compared to healthy teeth (Sukumar *et al.*, 2023).

It is noteworthy that ARGs, MRGs, and other lines of bacterial defense mechanisms predate the existence of synthetic antibiotics produced/used by humans. These adaptive traits have been shaped by billions of years of evolution since the appearance of the first bacteria (Figure S1). In addition to the mobile resistome, bacterial species possess a resistome consisting of genes in their single circular chromosome.

Surveillance of resistomes is a crucial tenet of the One Health initiative in combating antimicrobial resistance (Sukumar et al., 2023; WHO, 2023). One Health is an integrated, unifying approach to balance and optimize the health of people, animals, and the environment (WHO, 2023).

Table S2 provides examples of oral bacteria that have developed resistance to common antibiotics, such as β-lactam and Tetracycline, as well as other selected bacterial-resistance information.

### Host-defense peptides and other examples of the *Homo sapiens* response

There are known host-defense peptides (HDPs), originally described just as antimicrobial, but now have renewed significance as curators of the pervasive microbial loads required to maintain homeostasis and manage microbiome diversity (Meade and O’Farrelly, 2019). One of the best known and studied HDP families is the β-defensins which are produced in diverse combinations by epithelial and immune cells. β-defensins members, such as DEFB1, are peptides produced in diverse combinations by epithelial and immune cell populations, which manage the microbial colonization. Some alleles in polymorphic *DEFB1* loci are risk factors for PDs and caries (Table S3). 

Host cytokines also modulate the immune response, altering its efficiency in the competition against pathogens and increasing PDs susceptibility. Baker et al. (2021) found in the saliva of children with caries a significantly higher concentration of ten salivary immunological markers, such as interleukins (Baker *et al.*, 2021). Furthermore, some allele combinations in polymorphic loci in Interleukin-10 gene (single-nucleotide polymorphisms SNPs: IL10; A-1082G rs1800896, C-819T rs1800871, and C-592A rs1800872) may reduce IL-10 production and has been associated with oral pathologies, such as periodontitis. In contrast, other allele combinations promote the anti-inflammatory immune response, which is important for protection against the microbes that cause periodontitis (Lopes et al., 2017).

Considering large-scale genomic association studies (GWAS) and periodontitis or caries data, the first results were published more than 20 years ago (reviewed in Morelli et al., 2020). Other lines of research, including investigations with twins or families, and approaches with candidate genes, helped build a solid argument on the role of host genetic factors in oral diseases. For example, Schaefer et al. (2010) showed the *GLT6D1* SNP rs1537415 association with aggressive periodontitis. Bevilacqua et al. (2018) found association between the *CRACR2* (*EFCAB4B*) rs242016 and periodontitis in a small Italian population (Table S3 provides other examples). See also Morelli *et al.* (2020) and Weyrich (2021).

We compiled allele frequencies in human populations, considering selected genes with SNPs previously associated with PDs and/or caries like those mentioned above (Tables S3-S4). A statistical test revealed that the major geographical groups significantly differ in their risk/or protection alleles (Table S5), suggesting differential susceptibilities across human populations.

## Indigenous peoples, neglected peoples

The United Nations (UN) recognize that indigenous peoples are among the most vulnerable and disadvantaged groups in the world, emphasizing the need for special measures to protect their rights, preserve their unique cultures and ways of life, and address their health (UN, 2023). Despite this recognition, indigenous communities often remain invisible and marginalized, as exemplified by recent events, such as the Yanomami case in Brazil (Boadle, 2023). 

The scarcity of scientific studies on indigenous peoples is just one aspect of this invisibility problem. A 2009 survey found that 96% of participants in GWAS were Europeans or European descendants (Need and Goldstein, 2009). More recent data from Sirugo et al. (2019) showed that this bias remains strong, with 78% of people included in GWAS studies being Europeans or having European ancestry, only 10% being Asians or having Asian descent, 2% being Africans or having African descent, 1% being Latin Americans, and less than 1% being from other populations. This bias hinders the ability of the researchers to understand the genetic basis of common diseases with multifactorial inheritance (*e.g*., PDs and dental caries), which are characterized by many small-effect alleles whose frequencies can vary significantly across human populations. The lack of information from neglected and invisible populations prevents the use of methods for predicting polygenic genetic risk alleles for these common diseases, which affect millions of people from these populations worldwide. 

The same can be said for rare and Mendelian genetic diseases. Advances in genomic technologies are transforming the diagnosis of these diseases, but indigenous populations often experience inequities in diagnostic and therapeutic access (D’Angelo et al., 2020).

Without action to address these distortions, genomic medicine will soon be available only to a few privileged people (Bustamante et al., 2011; Popejoy and Fullerton, 2016).

Oral health is not exempt from health disparity trends among Indigenous populations; untreated dental caries, less restored teeth, and PDs have been reported to be higher in Indigenous than non-Indigenous populations (Nath et al., 2021). As a result, these neglected people experience amplified damage caused by worse oral health. 

The indigenous people of the American continent (or Native Americans) can be considered neglected regarding genetic/genomic and oral cavity health studies when they are compared with non-natives. Nevertheless, some efforts that resulted in publications already signal some trends. For example, it is known that the specific combination of social, historical, environmental, economic, and ethnic-cultural circumstances in which Indigenous and non-Indigenous individuals interact can create varying protective or risk factors that affect oral health outcomes in distinct ways for Indigenous groups (Arantes et al., 2021). 

The study performed by Arantes et al. (2021) found that Indigenous individuals from Guarani-Kaiowá, Guarani (unspecified regarding partiality), Terena, and Kadiwéu populations (Mato Grosso do Sul Brazilian State) had a lower incidence of tooth decay compared to non-Indigenous individuals, but they faced greater challenges in accessing restorative dental services.

Figueiredo et al. (2013) and Ribeiro et al. (2016) investigated a native community (Kiriri) in northeastern Brazil, whose members have been influenced by external sociocultural habits, including a diet rich in sugar, despite striving to preserve part of their traditions. The population has a high prevalence of destructive periodontal disease (98%), and individuals with this pathology had higher systolic blood pressure than controls (Ribeiro *et al.*, 2016).

Gaetti-Jardim et al. (2015) investigated the oral microbiota of individuals from the Umutina Indigenous Territory (IT), where an ancestral lifestyle is observed and there has been no admixture with non-native populations. The Umutina IT is home to 480 individuals of the Umutina, Paresi, Bororo, Bakairi, Kayabi, Irantxe, Nambikwara, and Terena peoples, whose occupation of the territory dates back at least six generations (Gaetti-Jardim *et al.*, 2015). The occurrence of pathogenic bacteria was similar to that previously described for other populations worldwide, but with some notable differences. The red complex *T. denticola* has a more restricted distribution in Native American populations, being observed in only 5% of the Guatemalan Maya and 13-17% of the natives of the Xingu IT, a number similar to that found in the Umutina IT (Gaetti-Jardim *et al.*, 2015). Likewise, *Actinobacillus actinomycetemcomitans* belonging to the green bacterial complex (Socransky and Haffajee, 2005), but currently recognized as one of the agents of PDs (Gholizadeh et al., 2017), had reduced prevalence in natives of the Umutina IT when compared to non-native Brazilians (Gaetti-Jardim *et al.*, 2015).

Clemente et al. (2015) characterized the oral bacterial microbiota of 34 individuals from an isolated Yanomami village. Their oral microbiota was compared with that found in urbanized non-native individuals living in the United States. The yellow bacteria genus *Streptococcus* dominated both populations, but the Yanomami had higher proportions of two other yellow genera, *Prevotella* and *Fusobacterium*. The authors found that despite any known exposure to antibiotics, bacteria in the Yanomami participants had ARGs that confer resistance to synthetic antibiotics. Clemente *et al.* (2015) emphasized the need for a broad characterization of the microbiota and its resistome, considering that indigenous peoples are still living according to ancestral lifestyles before modern practices promoted potential dysbiosis.

Lopes et al. (2017) investigated three *IL10* polymorphisms in urban non-indigenous individuals living in Belém (Pará, Brazilian State), and found that those with a higher contribution of Native American ancestral alleles had an increased risk of developing periodontitis.

Regarding other American countries, Ozga et al. (2016) used *16SrRNA* to access the microbiota of Cheyenne and Arapaho members living in Oklahoma (USA) and compared them with their non-indigenous neighbors. They found that the natives had a higher frequency of bacteria implicated in systemic disorders, such as those of the orange complex genus *Prevotella*. In another study, Agnello et al. (2017) sought to understand why Indigenous children in North America suffer with a higher degree of severe early caries (S-ECC) than the general population. They found that the S-ECC group had extremely higher level of cariogenic *Streptococcus mutans*, and 9-fold higher level of red complex genus *Porphyromonas* than the caries-free counterparts. 

Bravo-Lopez et al. (2020) reported the first direct evidence of the presence of red complex bacteria (*T*. *forsythia, P. gingivalis,* and *T. denticola*) in dental calculus samples from archaeological skeletons spanning from the pre-Columbian to the colonial period in Mexico. *T. forsythia* was the dominant bacteria, and the phylogenetic relationships of the strains showed that some of them arrived with the first migrants to America, while others arrived with Europeans and Africans in the 16th century. The authors also identified that the *tetQ* gene involved in resistance to Tetracycline was absent in all ancient *T. forsythia* strains.

Honap et al. (2023) investigated the skeletal remains of ancestors of the Wichita, a southern Plain Native American people from Oklahoma. They also found the presence of the three red complex species, regardless of oral disease, in the sampled tooth. The researchers corroborated that there were pre-Columbian strains of those bacteria, which were prevalent until Europeans arrived. 

Additional instances of indigenous peoples from other continents and ethical considerations regarding microbiota studies in these communities are available in the Supplementary Material section.

## Exploring bacterial complexes

Socransky et al. (1998) used a range of techniques available at the time of their studies, including DNA-DNA hybridization, to show contextual similarities between species within complexes and their relationship to clinical outcomes (Table S1). Basically, bacteria belonging to the blue, yellow, green, and purple complexes are linked to oral health. Conversely, bacteria from the orange and red complexes are associated with dysbiosis of the microbiota, most commonly leading to the development of diseases in the oral cavity and also in other organs and tissues of the body. 

**Figure 1 -:**
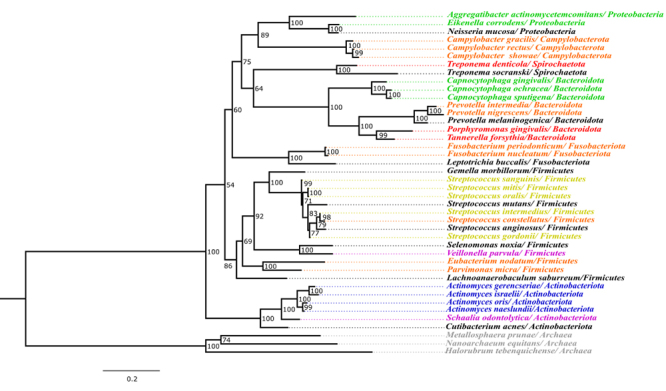
Maximum Likelihood phylogeny based on *16srRNA* sequences from 39 bacterial taxa. The colors in each taxon indicate the representation of complexes and groups. The Phyla nomenclature is according to the GTDB.

We have performed a phylogenetic analysis using *16SrRNA* sequences (see Additional information section/Material and Methods; Tables S6-S7) of the bacteria belonging to these complexes, and the topology is present in Figure 1. The tree illustrates that the species in the complexes do not necessarily cluster together. Figure S2 shows a network with the same data set, another way to illustrate the differences and similarities. This result may be indicating some level of evolutionary convergence that leads bacteria not closely related phylogenetically to have similar characteristics (*e.g*., virulence or non-virulence), when the consortium between them confers an adaptive advantage under others and the host. For instance, the three main members of the red complex, *T. forsythia, P. gingivalis,* and *T. denticola* belong to two different phyla, predicted by HOMD and GTDB consortium methodologies (Table S1), and are equally capable of producing various virulence factors that assist in their survival and contribute to the development and progression of the oral disease in the host: *P. gingivalis* produces, for example, heat shock proteins, while *T. forsythia* and *T. denticola* utilize proteases and dentilisin, respectively, among others, as virulence factors. *In vivo* studies have shown that these bacteria have nutritional interdependency, and the ability to regulate the virulence factors of each other (Nayak et al., 2018). These phyla are separated by about 2.8 billion years (Figure S1).

Noteworthy, the notion of complexes to indicate the level of periodontal health and pathogenicity, is being challenged. For example, in addition to the primary members of the red and orange complexes, there are other bacteria that have been suggested to be involved in PDs. These include: *Filifactor alocis, Fretibacterium fastidiosum, Eikenella corrodens, Capnocytophaga* spp*.,* and *Aggregatibacter actinomycetemcomitans*, the last three genera already associated with the yellow and green complexes, respectively, whose members are more commonly associated with periodontal health contexts (Gholizadeh et al., 2017; Kang et al., 2021; Ozuna et al., 2022; Pandian et al., 2023). Kang *et al.* (2021) concluded that beyond the red complex *T. forsythia* and the orange complex *C. rectus*, *F. fastidiosum* is also a key driver during microbiota alteration in the progression of periodontitis. 

Valdebenito et al. (2018) analyzed the effects of competition between two prevalent species in individuals with healthy teeth (*Streptococcus sanguinis;* Yellow complex) or with caries (*Streptococcus mutans*). These two species occupy the same ecological site and have similar metabolic needs. The study focused on characterizing the differences in their genomes to explore the potential genetic advantages of one over the other, since they are antagonist species. For instance, *S. sanguinis* produces hydrogen peroxide (H_2_O_2_; gene *spxB*) with antimicrobial activity, but this bacterium has also an enzymatic machinery involved in H_2_O_2_ detoxification. *S. mutans* itself does not produce significant amounts of H_2_O_2_, and it is highly susceptible to H_2_O_2_ because it lacks an efficient detoxification machinery. The authors found that *S. sanguinis* has three additional detoxification enzymes, which could be advantageous over *S. mutans*. In contrast, mutacin is involved in a system responding to environmental changes, including the ability to incorporate foreign DNA (competence) and biofilm development. Mutacin allows *S. mutans* to adapt and survive under stress (extremely acidic conditions), an unfavorable condition for *S. sanguinis*. This example illustrates that even within the same genus, there are bacteria associated with a healthy state and those related to oral pathology, indicating an antagonistic condition, which potentially triggers a systematic arms race or Red Queen dynamics between them. Interestingly, *S. sanguinis* is predominantly isolated in healthy children, whereas non-detectable levels were found in individuals with carious lesions, suggesting a replacement. In this case, the Black Queen hypothesis loses strength, at least considering the evolutionary relationships between these two streptococcal species. 

## Conclusions

General microbiota patterns are observed in healthy individuals and those with oral pathologies, although with some individual variation. Microbiota variability is also notable among human populations. Bacteria from different phyla can also form adaptive consortia, while those from the same genus can result in different outcomes for the host, indicating antagonism. Furthermore, many lines of evidence reinforce the idea that the genetic profile of the human host is related to the dynamics of its oral microbiota and vice versa (Weyrich, 2021). 

The influence of host and microbiota genetics, evolution, environmental exposures, treatments, and bacterial resistance to antibiotic stresses the need to consider the problem within multidisciplinary or even translational approaches, such as the One Health initiative proposed by World Health Organization. 

Despite the challenges faced by this complex theme, reversing the global trend of deaths and loss of quality of life caused by oral infectious diseases, particularly in countries with low human development indices and neglected populations, is essential.

We conclude with the hope that in a future review it will no longer be necessary to refer to Native American peoples as neglected groups when compared to others.

## Supplementary material

Supplementary Material - the following online material is available for this article:

Table S1 - Species belonging to bacterial complexes used for the phylogenetic analysis.

Table S2 - Examples of oral bacteria with strains resistant to common antibiotics.

Table S3 - Examples of SNPs associated with caries and/or PDs.

Table S4 - Selected SNPs associated to the PDs and/or caries and allele frequencies.

Table S5 - χ²statistical test calculated for major human groups based on allele frequencies.

Table S6 - Bacterial species analyzed for the *16SrRNA* gene.

Table S7 - Microbial composition of the supra and subgingival plaque classified by complex..

Figure S1 - Divergence time tree considering 39 bacterial taxa.

Figure S2 -
*16srRNA* sequence-based haplotype network considering 39 bacterial taxa.

Additional information regarding the main topics of the review.
