# Supplementary material for: Oral microbiota, co-evolution, and implications for health and disease: The case of indigenous peoples
Source: Genet Mol Biol. 2024 Jan 22;46(3 Suppl 1):e20230129. doi: 10.1590/1678-4685-GMB-2023-0129 (PMC10829892; doi:10.1590/1678-4685-GMB-2023-0129)
Supplement: Table S1 - [file 1415-4757-GMB-46-03-s1-e20230129-s1.pdf]

*Genetics and Molecular Biology*

https://doi.org/10.1590/1678-4685-GMB-2023-0129

# Supplementary Material to "Oral microbiota, co-evolution, and implications for health and disease: the case of indigenous peoples"

**Table S1** - Species belonging to Bacterial complexes and others used for the phylogenetic analysis with *16SrRNA* sequences.

| Taxonomic classification | | | Complex classification† | References |
|---|---|---|---|---|
| **Phylum** | **Family** | **Species** | | |
| Bacteroidetes‡<br>Bacteroidota§ | Porphyromonadaceae | *Tannerella forsythia*¶<br>*(Bacteroides forsythus[1])* | Red | [1]Socransky *et al.* (1998) |
| Spirochaetes‡<br>Spirochaetota§ | Treponemataceae | *Treponema denticola* | Red | Socransky *et al.* (1998) |
| Bacteroidetes‡<br>Bacteroidota§ | Porphyromonadaceae | *Porphyromonas gingivalis* | Red | Socransky *et al.* (1998) |
| Firmicutes | Peptoniphilaceae | *Parvimonas micra*¶<br>*(Peptostreptococcus micros[1])* | Orange | [1]Socransky *et al.* (1998) |
| Firmicutes | Streptococcaceae | *Streptococcus constellatus* | Orange | Socransky *et al.* (1998) |
| Firmicutes | Peptostreptococcaceae‡<br>Anaerovoracaceae§ | *Eubacterium nodatum*[1,‡]<br>*Hornefia nodata*§ | Orange | [1]Socransky *et al.* (1998) |
| Proteobacteria‡<br>Campylobacterota§ | Campylobacteraceae | *Campylobacter gracilis* | Orange | Socransky *et al.* (1998) |
| Proteobacteria‡<br>Campylobacterota§ | Campylobacteraceae | *Campylobacter rectus* | Orange | Socransky *et al.* (1998) |
| Proteobacteria‡<br>Campylobacterota§ | Campylobacteraceae | *Campylobacter showae* | Orange | Socransky *et al.* (1998) |

| Taxonomic classification | | | Complex classification[†] | References |
|---|---|---|---|---|
| **Phylum** | **Family** | **Species** | | |
| Bacteroidetes[‡] Bacteroidota[§] | Prevotellaceae | *Prevotella intermedia* | Orange | Socransky *et al.* (1998) |
| Bacteroidetes[‡] Bacteroidota[§] | Prevotellaceae | *Prevotella nigrescens* | Orange | Socransky *et al.* (1998) |
| Fusobacteria[‡] Fusobacteriota[§] | Fusobacteriaceae | *Fusobacterium periodonticum* | Orange | Socransky *et al.* (1998) |
| Fusobacteria[‡] Fusobacteriota[§] | Fusobacteriaceae | *Fusobacterium nucleatum ss nucleatum*[1] *Fusobacterium nucleatum*[‡,§] | Orange | [1]Socransky *et al.* (1998) |
| Fusobacteria[‡] Fusobacteriota[§] | Fusobacteriaceae | *Fusobacterium nucleatum ss vincentii*[1,‡] *Fusobacterium vincentii*[§] | Orange | [1]Socransky *et al.* (1998) |
| Fusobacteria[‡] Fusobacteriota[§] | Fusobacteriaceae | *Fusobacterium nucleatum ss polymorphum*[1,‡] *Fusobacterium polymorphum*[§] | Orange | [1]Socransky *et al.* (1998) |
| Firmicutes | Streptococcaceae | *Streptococcus mitis* | Yellow | Socransky *et al.* (1998) |
| Firmicutes | Streptococcaceae | *Streptococcus oralis* | Yellow | Socransky *et al.* (1998) |
| Firmicutes | Streptococcaceae | *Streptococcus gordonii* | Yellow | Socransky *et al.* (1998) |
| Firmicutes | Streptococcaceae | *Streptococcus intermedius* | Yellow | Socransky *et al.* (1998) |
| Firmicutes | Streptococcaceae | *Streptococcus sanguinis* | Yellow | Socransky *et al.* (1998) |
| Bacteroidetes[‡] Bacteroidota[§] | Flavobacteriaceae | *Capnocytophaga gingivalis* | Green | Socransky *et al.* (1998) |
| Bacteroidetes[‡] Bacteroidota[§] | Flavobacteriaceae | *Capnocytophaga ochracea* | Green | Socransky *et al.* (1998) |
| Bacteroidetes[‡] Bacteroidota[§] | Flavobacteriaceae | *Capnocytophaga sputigena* | Green | Socransky *et al.* (1998) |
| Proteobacteria | Pasteurellaceae | *Aggregatibacter actinomycetemcomitans*[¶] *(Actinobacillus actinomycemcomitans*[1]*)* | Green | [1]Socransky *et al.* (1998) |
| Proteobacteria | Neisseriaceae | *Eikenella corrodens* | Green | Socransky *et al.* (1998) |

| Taxonomic classification | | | Complex classification[†] | References |
|---|---|---|---|---|
| Phylum | Family | Species | | |
| Proteobacteria[‡]<br>Campylobacterota[§] | Campylobacteraceae | *Campylobacter concisus* | NC[1]<br>Green[2,3] | [1]Socransky *et al.* (1998)<br>[2]Carrouel *et al.* (2016)<br>[3]Gambin *et al.* (2021) |
| Firmicutes | Veillonellaceae | *Veillonella parvula* | Purple | Socransky *et al.* (1998) |
| Actinobacteria[‡]<br>Actinobacteriota[§] | Actinomycetaceae | *Actinomyces odontolyticus*[1]<br>*Schaalia odontolytica*[‡]<br>*Pauljensenia odontolytica*[§] | Purple | [1]Socransky *et al.* (1998) |
| Actinobacteria[‡]<br>Actinobacteriota[§] | Actinomycetaceae | *Actinomyces viscosus*[¶]<br>*(Actinomyces naesludii genospecies 2*[1]*)* | NC[1]<br>Blue[2,3] | [1]Socransky *et al.* (1998)<br>[2]Socransky and Haffajee (2002)<br>[3]Uzel *et al.* (2011) |
| Actinobacteria[‡]<br>Actinobacteriota[§] | Actinomycetaceae | *Actinomyces gerencseriae* | NC[1]<br>Blue[2,3] | [1]Socransky *et al.* (1998)<br>[2]Socransky and Haffajee (2002)<br>[3]Uzel *et al.* (2011) |
| Actinobacteria[‡]<br>Actinobacteriota[§] | Actinomycetaceae | *Actinomyces israelii* | NC[1]<br>Blue[2,3] | [1]Socransky *et al.* (1998)<br>[2]Socransky and Haffajee (2002)<br>[3]Uzel *et al.* (2011) |
| Actinobacteria[‡]<br>Actinobacteriota[§] | Actinomycetaceae | *Actinomyces oris* | NC[1]<br>Blue[2,3] | [1]Socransky *et al.* (1998)<br>[2]Socransky and Haffajee (2002)<br>[3]Uzel *et al.* (2011) |
| Actinobacteria[‡]<br>Actinobacteriota[§] | Propionibacteriaceae | *Propionibacterium acnes*<br>*Cutibacterium acnes*[‡,§] | NC | Ximénez-Fyvie *et al.* (2000) |
| Firmicutes | Selenomonadaceae | *Selenomonas noxia*[‡]<br>*Centipeda noxia*[§] | NC | Socransky *et al.* (1998) |
| Firmicutes | Lachnospiraceae | *Lachnoanaerobaculum saburreum*<br>*(Eubacterium sabureum*[4]*)* | NC | [4]Ximénez-Fyvie *et al.* (2000) |
| Firmicutes | Gemellaceae | *Gemella morbillorum* | NC | Ximénez-Fyvie *et al.* (2000) |

| Taxonomic classification | | | Complex classification† | References |
|---|---|---|---|---|
| Phylum | Family | Species | | |
| Fusobacteria‡ Fusobacteriota§ | Leptotrichiaceae | *Leptotrichia buccalis* | NC | Ximénez-Fyvie *et al.* (2000) |
| Proteobacteria | Neisseriaceae | *Neisseria mucosa* | NC | Ximénez-Fyvie *et al.* (2000) |
| Bacteroidetes‡ Bacteroidota§ | Prevotellaceae‡ Bacteroidaceae§ | *Prevotella melaninogenica* | NC | Ximénez-Fyvie *et al.* (2000) |
| Firmicutes | Streptococcaceae | *Streptococcus anginosus* | NC | Ximénez-Fyvie *et al.* (2000) |
| Firmicutes | Streptococcaceae | *Streptococcus mutans* | NC | Uzel *et al.* (2011) |
| Spirochaetes‡ Spirochaetota§ | Treponemataceae | *Treponema socranskii* | NC | Ximénez-Fyvie *et al.* (2000) |
| Firmicutes | Staphylococcaceae | *Staphylococcus aureus* | NC | Cheung *et al.* (2021) |

Bacterial species belonging to the blue, yellow, green, and purple complexes are linked to oral health. Conversely, bacterial species from the orange and red complexes are associated with dysbiosis of the microbiota, leading to the development of periodontal diseases and systemic diseases. NC = No classification according to complexes. *Staphylococcus aureus* did not enter the phylogenetic analysis but is cited in the main text. Note that † are the bacterial complexes suggested by Socransky *et al.* (1998) and expanded in Ximénez-Fyvie *et al.* (2000, to include the blue complex); ‡ is the taxonomic name used in the Human Oral Microbiome Database (HOMD, March 2023) V3.1; § is the taxonomic name used in Genome Taxonomic Database (GTDB, April 2022); ¶ is the for the bacteria whose names were changed up to 2022. According to the International Code of Nomenclature of Prokaryotes (ICNP) 2022 revision (Oren *et al.*, 2022), phylum name is the name of the designated type genus plus the suffix -ota, however, exceptions regarding it can be made by the Judicial Commission.

## References


Carrouel F, Viennot S, Santamaria J, Veber P and Bourgeois D (2016) Quantitative molecular detection of 19 major pathogens in the interdental biofilm of periodontally healthy young adults. Front Microbiol 7:840.

Cheung GY, Bae JS and Otto M (2021) Pathogenicity and virulence of Staphylococcus aureus. Virulence 12:547-569.

Gambin DJ, Vitali FC, De Carli JP, Mazzon RR, Gomes BP, Duque TM and Trentin MS (2021) Prevalence of red and orange microbial complexes in endodontic-periodontal lesions: A systematic review and meta-analysis. Clin Oral Investig 25:6533-6546.

Oren A, Arahal DR, Göker M, Moore ERB, Rossello-Mora R and Sutcliffe IC (2022) International Code of Nomenclature of Prokaryotes. Prokaryotic Code (2022 Revision). Int. J. Syst. Evol. Microbiol. 73:005585

Socransky SS and Haffajee AD (2002) Dental biofilms: Difficult therapeutic targets. Periodontol 2000 28:12-55.

Socransky SS, Haffajee AD, Cugini MA, Smith CKJR and Kent Jr RL (1998) Microbial complexes in subgingival plaque. J Clin Periodontol 25:134-144.

Uzel NG, Teles FR, Teles RP, Song XQ, Torresyap G, Socransky SS and Haffajee AD (2011) Microbial shifts during dental biofilm re-development in the absence of oral hygiene in periodontal health and disease. J Clin Periodontol 38:612-620.

Ximénez-Fyvie LA, Haffajee AD and Socransky SS (2000) Comparison of the Microbiota of supra-and subgingival plaque in health and periodontitis. J Clin Periodontol 27:648-657.

**Internet Resources**

Human Oral Microbiota Database (HOMD) (2023) Human Oral Microbiota Database v. 3.1, https://www.homd.org/ (accessed 8 and 14 February 2022; accessed April 12th and 19th 2022).

Genome Taxonomic Database (2023) Welcome to GTDB, http://gtdb.ecogenomic.org/ (accessed 14 April 2023).
