# Supplementary material for: Oral microbiota, co-evolution, and implications for health and disease: The case of indigenous peoples
Source: Genet Mol Biol. 2024 Jan 22;46(3 Suppl 1):e20230129. doi: 10.1590/1678-4685-GMB-2023-0129 (PMC10829892; doi:10.1590/1678-4685-GMB-2023-0129)
Supplement: Table S2 - [file 1415-4757-GMB-46-03-s1-e20230129-s2.pdf]

Genetics and Molecular Biology

https://doi.org/10.1590/1678-4685-GMB-2023-0129

Supplementary Material to "Oral microbiota, co-evolution, and implications for health and disease: the case of indigenous peoples"

Table S2 - Examples of oral bacteria where strains resistant to two classes of common antibiotics have already been identified[1].

Gram negative bacteria present in the oral cavity where strains resistant to β-lactam antibiotics[2] have already been described	Gram negative bacteria present in the oral cavity where strains resistant to Tetracyclines[3] have already been described
Eikenella corrodens	*Aggregatibacter actinomycetemcomitans*
Haemophilus influenzae	*Capnocytophaga ochracea*
Haemophilus influenzae	*Eikenella corrodens*
Haemophilus parainfluenzae	*Fusobacterium nucleatum*
Haemophilus paraphrohaemolyticus	*Neisseria perflava/sicca*
Fusobacterium nucleatum	*Neisseria meningitidis*
Porphyromonas gingivalis	*Neisseria mucosa*
	Porphyromonas gingivalis
	Treponema denticola
	Prevotella intermedia
	Prevotella nigrescens
Gram positive bacteria present in the oral cavity where strains resistant to β-lactam antibiotics[2] have already been described	Gram positive bacteria present in the oral cavity where strains resistant to Tetracyclines[3] have already been described
Staphylococcus aureus	*Actinomyces viscosus*
	Corynebacterium striatum
	Parvimonas micra
	Staphylococcus aureus
	Streptococcus agalactiae
	Streptococcus anginosus
	Streptococcus bovis
	Streptococcus canis
	Streptococcus constellatus
	Streptococcus gordonii
	Streptococcus mitis
	Streptococcus mutans

	Streptococcus milleri
	Streptococcus oralis
	Streptococcus parasanguis
	Streptococcus pneumoniae
	Streptococcus pyogenes
	Streptococcus salivarius
	Streptococcus sanguinis

[1]Based on Roberts (1998) the upper respiratory/oral tract is entirely covered with bacteria, which are present on the mucosal surfaces, gingiva, tooth surfaces, and in between the gingiva and teeth. However, the microflora is not a fixed entity and undergoes changes in its composition over time, responding to various intrinsic and extrinsic factors of the individual. Recent clinical evidence suggests that 19.6% of samples taken from oral and maxillofacial infections contain multidrug-resistant bacteria. Furthermore, respiratory, and oral bacteria are continuously evolving to develop resistance to antibiotics. Recent research has identified a soluble bacterial lipocalin that captures various antibiotics at infection sites distant from its original location (Jiang *et al.*, 2018). Antibiotic resistance is a significant concern for human health, and it stands as one of the rare instances where we can observe evolution happening in real-time (Martinez, 2014).

According to Sukumar *et al.* (2016) antibiotic resistance can arise through two main mechanisms: Intrinsic and acquired. Intrinsic resistance is a natural characteristic of a bacterial species that makes it inherently resistant to certain antibiotics. An example of intrinsic resistance is the resistance of Escherichia coli to vancomycin. In this case, the vancomycin molecule is too large to pass through the porin channels in the outer membrane of the *E. coli* cell wall, rendering the antibiotic ineffective against the bacteria. On the other hand, acquired resistance results from horizontal gene transfer (HGT) or mutation, where bacteria acquire resistance genes from other organisms or undergo genetic changes that confer resistance to antibiotics. This can happen through mechanisms such as conjugation, transduction, and transformation. It is important to understand the mechanisms of antibiotic resistance in order to develop effective strategies for combating the spread of antibiotic-resistant bacteria. By identifying the underlying causes of resistance, researchers can develop new antibiotics that target resistance bacteria or explore alternative treatment option.

[2]β-lactam antibiotics inhibit bacterial cell wall biosynthesis. Bacterial strains have developed resistance to β-lactam antibiotics primarily through the action of β-lactamases, which are enzymes that break down the β-lactam ring in the antibiotic molecule. This renders the antibiotic ineffective against the bacteria's penicillin-binding proteins, which are the drug's target (Ho *et al.*, 2019). Arredondo *et al.* (2020) investigated subgingival samples from 130 patients diagnosed with generalized periodontitis. The researchers cultured the samples on agar plates containing either amoxicillin or cefotaxime. They found that 83% of the patients harbored β-lactam-resistant isolates. They found 35 different genera, with *Prevotella* and *Streptococcus* being the most frequently identified genera. The *blaCfxA* was the most prevalent ARG, present in 24.8% of the isolates, followed by *blaTEM* (12.9%). Noteworthy, that the efficiency of β-lactam antibiotics has been increased by the association with β-lactamase enzyme inhibitors such as Clavulanic acid (Zango *et al.*, 2019).

[3]Tetracyclines inhibit bacterial protein synthesis by attaching to the site A of the 30S component of the ribosome. Tetracyclines does not kill the bacteria but stops its multiplication (Ademikanra *et al.*, 2023 and references therein). Bacteria can acquire resistance to Tetracyclines primarily through four main mechanisms: (1) efflux, where the bacteria pump tetracycline forcefully into their cytoplasm against a concentration gradient;(2) ribosome protection proteins,

which bind to the ribosome and remove the drug from its target site; (3) enzymatic resistance, where the bacteria produce enzymes that modify or degrade the antibiotic; and (4) mutations in the *16SrRNA*, which reduce the binding affinity of the drug to the ribosome. There are multiple genes of each of these mechanisms identified so far (Nguyen *et al.*, 2014; Ademikanra *et al.*, 2023). For example, Villedieu *et al.* (2003) collected oral samples from 20 healthy adults to identify Tetracycline-resistant bacteria and their antimicrobial resistance genes (ARGs). The majority of resistant isolates carried ARGs encoding ribosomal protection proteins, with *tet(M)* being the most commonly identified ARG. Others included *tet(W)* (21%), *tet(O), tet(Q)* (10.5% and 9.5% of isolates), and *tet(S)* (2.8% of isolates). Efflux protein-encoding genes were found in 4.8% of Tetracycline-resistant isolates, with *tet(L)* present in 2.8% of isolates, and *tet(A)* and *tet(K)* in 1% of isolates each. The study by Villedieu *et al.* (2003), published 20 years ago, showed for the first time in oral bacteria the gene *tet(W*) and that a variety of Tetracycline ARGs were present in the oral microbiota of healthy adults.

The use of β-lactam and Tetracycline antibiotics, two of the most widely prescribed classes of antibacterial agents, makes them powerful natural selection agents for bacteria strains with advantageous mutations in genes (ARGs) encoding proteins involved in their survival and reproduction protection mechanisms.

References

Ademikanra AF, Oyewole OM, Olayiwola AO and Areo SO (2023) Tetracycline resistance on protein synthesis: A brief review. Biomed Chem Sci 2:95-101.

Arredondo A, Blanc V, Mor C, Nart J and León R (2020) Resistance to β-lactams and distribution of β-lactam resistance genes in subgingival microbiota from Spanish patients with periodontitis. Clin Oral Invest 24:4639-4648.

Ho S, Nguyen L, Trinh T and MacDougall C (2019) Recognizing and overcoming resistance to new beta-lactam/beta-lactamase inhibitor combinations. Curr Infect Dis Rep 21:39.

Jiang S, Zeng J, Zhou X and Li Y (2018) Drug resistance and gene transfer mechanisms in respiratory/oral bacteria. J Dent Res 97:1092-1099.

Martinez JL (2014) General principles of antibiotic resistance in bacteria. Drug Discov Today Technol 11:33-39.

Nguyen F, Starosta AL, Arenz S, Sohmen D, Dönhöfer A and Wilson DN (2014) Tetracycline antibiotics and resistance mechanisms. Biol Chem 395:559-575.

Roberts MC (1998) Antibiotic resistance in oral/respiratory bacteria. Crit Rev Oral Biol Med 9:522-540.

Sukumar S, Roberts AP, Martin FE and Adler CJ (2016) Metagenomic insights into transferable antibiotic resistance in oral bacteria. J Dent Res 95:969-976.

Villedieu A, Diaz-Torres ML, Hunt N, McNab R, Spratt DA, Wilson M and Mullany P (2003) Prevalence of tetracycline resistance genes in oral bacteria. Antimicrob Agents Chemother 47:878-882.

Zango UU, Ibrahim M, Shawai SAA and Shamsuddin IM (2019) A review on β-lactam antibiotic drug resistance. MOJ Drug Des Develop Ther 3:52-58.
