# Supplementary material for: Oral microbiota, co-evolution, and implications for health and disease: The case of indigenous peoples
Source: Genet Mol Biol. 2024 Jan 22;46(3 Suppl 1):e20230129. doi: 10.1590/1678-4685-GMB-2023-0129 (PMC10829892; doi:10.1590/1678-4685-GMB-2023-0129)
Supplement: Table S3 - [file 1415-4757-GMB-46-03-s1-e20230129-s3.pdf]

Genetics and Molecular Biology

https://doi.org/10.1590/1678-4685-GMB-2023-0129

Supplementary Material to "Oral microbiota, co-evolution, and implications for health and disease: the case of indigenous peoples"

Table S3 - Examples of SNPs associated with caries and/or PDs.

Gene and main role	RS ID[1]	Gene consequence	Change (GRCh37.p13)	Potential risk allele	Associated phenotype	Population/ Country/ Continent	References of risk allele and population
DEFB1 Antibacterial, antiviral and antifungal activities.	rs1799946	5 Prime UTR Variant	NC_000008.10:g.6735431C>T	T	Higher DMFT[2] index	North-Eastern Italy	Navarra *et al.* (2016)
				T	Dental caries susceptibility	China	Wu *et al.* (2022)
				T	Persistent apical periodontitis (PAP)	Brazil	Antunes *et al.* (2021)
				T	Periodontitis risk	Asia	Chen *et al.* (2019)
	rs1800972	5 Prime UTR Variant	NC_000008.10:g.6735423C>G,T	G	Risk factor for Aggressive periodontitis (AP).	General population (meta-analysis)	Chen *et al.* (2019)
				G	Periodontitis and severe chronic periodontitis	Japan	Ikuta *et al.* (2015)
				C	Chronic periodontitis	North-eastern Italy	Zupin *et al.* (2017)
	rs11362	5 Prime UTR Variant		T	Higher risk of caries	Brazil / China	De Oliveira *et al.* (2018) / Ma *et al.* (2023) and

			NC_000008.10:g.6735399C>G,T				Wu *et al.* (2022)
				T	Higher incidence of caries in permanent dentition	General population (meta-analysis)	Hatipoğlu and Saydam (2020)
				T	Periodontitis risk	Asia	Chen *et al.* (2019)
				C	Higher DMFT[1] index	North-Eastern Italy	Navarra *et al.* (2016)
				C	Chronic periodontitis	Brazil/ North-eastern Italy	Dias *et al.* (2018) / Zupin *et al.* (2017)
				T	Significantly lower risk of developing PAP	Brazil	Antunes *et al.* (2021)
	rs1047031	3 Prime UTR Variant	NC_000008.10:g.6728198C>T	T	Chronic periodontitis and AP	Caucasian[5]	Schaefer *et al.* (2010)
MUC5B Lubrication, pellicle formation, antimicrobial defense, and water retention.	rs2735733	Intron Variant	NC_000011.9:g.1261640C>A,T	T	Dental caries	Brazil	Cavallari *et al.* (2018)
	rs2249073		NC_000011.9:g.1273833T>C	C	Dental caries	Brazil	Cavallari *et al.* (2018)
	rs2857476		NC_000011.9:g.1281134T>C	T	Dental caries	Brazil	Cavallari *et al.* (2018)
LTF	rs1126477	Missense Variant	NC_000003.11:g.46501268C>T	T	Slight periodontitis	North-Eastern Italy	Zupin *et al.* (2017)

Modulates the host immune-inflammatory and antibacterial response.	rs1126478		NC_000003.11:g.46501213T>A,C,G	G	Higher risk of chronic periodontitis	North-Eastern Italy	Zupin *et al.* (2017)
	rs6441989	none	NC_000003.11:g.46474899A>C,G,T	A[4]	Protective effect against caries	Brazil	Doetzer *et al.* (2015)
IL-10<br><br>Key moderator of inflammation. Stimulates the production of protective antibodies and down-regulates pro-inflammatory cytokines.	rs1800896	*IL-19* : Intron Variant<br><br>*IL-10* : 2KB Upstream Variant	NC_000001.10:g.206946897T>C	C	Generalized aggressive form of periodontitis development	Czech Republic	Borilova Linhartova *et al.* (2020)
				C	Chronic periodontitis	China	Li *et al.* (2019)
				T	Chronic periodontitis	Caucasian[5]	Li *et al.* (2019)
	rs1800871		NC_000001.10:g.206946634A>G	A	Chronic periodontitis	Indians	Majumder *et al.* (2019)
	rs1800872		NC_000001.10:g.206946407T>G	T	Chronic periodontitis	Latinos[5]	Zhang *et al.* (2019)
				G	Chronic periodontitis	Indians	Majumder *et al.* (2019)
CRACR2 [3]<br><br>Involved in Ca2+ regulation during inflammation. Largely expressed in minor salivary glands.	rs242016	Synonymous Variant	NC_000012.11:g.3788260G>A	A	Localized periodontitis	Italia (isolated villages)	Bevilacqua *et al.* (2018)

[1] Reference Single Nucleotide Polymorphism Identification. [2] Decayed, missing due to caries, and filled teeth in the permanent teeth. [3] Also called, *EFCAB4B*. [4] Not considered as a risk allele, considered as a protective allele. [5]Population classification as cited by authors.

References

Antunes LS, Carvalho L, Petean IBF, Antunes LA, Freitas JV, Salles AG, Olej B, Oliveira DSB, Küchler EC and Sousa-Neto MD (2021) Association between genetic polymorphisms in the promoter region of the defensin beta 1 gene and persistent apical periodontitis. Int Endod J 54:38-45.

Bevilacqua L, Navarra CO, Pirastu N, Lenarda R Di, Gasparini P and Robino A (2018) A genome-wide association study identifies an association between variants in EFCAB4B gene and periodontal disease in an Italian isolated population. J Periodontal Res 53:992-998.

Borilova Linhartova P, Danek Z, Deissova T, Hromcik F, Lipovy B, Szaraz D, Janos J, Fassmann A, Bartova J, Drizhal I *et al.* (2020) Interleukin gene variability and periodontal bacteria in patients with generalized aggressive form of periodontitis. Int J Mol Sci 21:4728.

Cavallari T, Salomão H, Moysés ST, Moysés SJ and Werneck RI (2018) The impact of MUC 5B gene on dental caries. Oral Dis 24:372-376.

Chen C, Fan X, Yu S, Liu P, Pan Y, Lin L and Li C (2019) Association between Periodontitis and gene polymorphisms of hBD-1 and CD14: A meta-analysis. Arch Oral Biol 104:141-149.

De Oliveira DSB, Segato RAB, Oliveira S, Dutra ALT, Santos AS dos, Praxedes ADN, Belém LC, Antunes LA, Lips A, Nelson-Filho P *et al.* (2018) Association between genetic polymorphisms in DEFB1 and microRNA202 with caries in two groups of Brazilian children. Arch Oral Biol 92:1-7.

Dias RSAM, Pinho RCM, Almeida FR, Bandeira FAF, Da Silva RC, Crovella S, Farias BCF and Cimões R (2018) Evaluation of DEFB1 polymorphisms in individuals with chronic periodontitis and diabetes mellitus type 2 in a population of northeastern Brazil. Spec Care Dentist 38:227-233.

Doetzer AD, Brancher JA, Pecharki GD, Schlipf N, Werneck R, Mira MT, Riess O, Bauer P and Trevilatto PC (2015) Lactotransferrin gene polymorphism associated with caries experience. Caries Res 49:370-377.

Hatipoğlu Ö and Saydam F (2020) Association between rs11362 polymorphism in the beta-defensin 1 (DEFB1) gene and dental caries: A meta-analysis. J Oral Biosci 62:272-279.

Ikuta T, Inagaki Y, Tanaka K, Saito T, Nakajima Y, Bando M, Kido J and Nagata T (2015) Gene polymorphism of β-defensin-1 is associated with susceptibility to periodontitis in Japanese. Odontology 103:66-74.

Li Y, Hu B, Feng G, Chen Q, Zhu M, Ying S and Song J (2019) Association of interleukin-10-1082 (-1087) A> G polymorphisms and periodontitis risk: An updated meta-analysis based on 26 case-control studies. Ann Hum Genet 83:299-309.

Ma F, He H, Chen S, Yu X, Liu Q and Zeng X (2023) Associations of PART1 and DEFB1 polymorphisms with Dental Caries in twelve-year-old children in Southern China: A cross-sectional study. BMC Pediatr 23:6.

Majumder P, Panda SK, Ghosh S and Dey SK (2019) Interleukin gene polymorphisms in chronic periodontitis: A case-control study in the Indian population. Arch Oral Biol 101:156-164.

Navarra CO, Robino A, Pirastu N, Bevilacqua L, Gasparini P, Di Lenarda R and Crovella S (2016) Caries and innate immunity: DEFB1 gene polymorphisms and caries susceptibility in genetic isolates from North-Eastern Italy. Caries Res 50:589-594.

Schaefer AS, Richter GM, Nothnagel M, Laine ML, Rühling A, Schäfer C, Cordes N, Noack B, Folwaczny M, Glas J *et al.* (2010) A 3′ UTR transition within DEFB1 is associated with chronic and aggressive periodontitis. Genes Immun 11:45-54.

Wu L, Li Z, Zhou J, Ma B, Yu F, Zheng X, Hu X, Ma Z and Su X (2022) An association analysis for genetic factors for dental caries susceptibility in a cohort of Chinese children. Oral Dis 28:480-494.

Zhang Z, Zheng Y and Li X (2019) Interleukin-10 gene polymorphisms and chronic periodontitis susceptibility: Evidence based on 33 studies. J Periodontal Res 54:95-105.

Zupin L, Robino A, Navarra C, Pirastu N, Di Lenarda R, Gasparini P, Crovella S and Bevilacqua L (2017) LTF and DEFB1 polymorphisms are associated with susceptibility toward chronic periodontitis development. Oral Dis 23:1001-1008.
