# Supplementary material for: Oral microbiota, co-evolution, and implications for health and disease: The case of indigenous peoples
Source: Genet Mol Biol. 2024 Jan 22;46(3 Suppl 1):e20230129. doi: 10.1590/1678-4685-GMB-2023-0129 (PMC10829892; doi:10.1590/1678-4685-GMB-2023-0129)
Supplement: Table S4 - [file 1415-4757-GMB-46-03-s1-e20230129-s4.pdf]

Genetics and Molecular Biology

https://doi.org/10.1590/1678-4685-GMB-2023-0129

Supplementary Material to "Oral microbiota, co-evolution, and implications for health and disease: the case of indigenous peoples"

Table S4 - Frequency of SNPs associated with caries or periodontitis by population.

Gene	rs	Population	Ref	ALT	Sample size	Reference
DEFB1	rs1800972	Global	C=0.171616	G=0.828384	264690	TopMed
		American	C=0.264	G=0.736	980	1000Genomes_30x
		African	C=0.0476	G=0.9524	1786	1000Genomes_30x
		European	C=0.2070	G=0.7930	1266	1000Genomes_30x
		East Asian	C=0.0957	G=0.9043	1170	1000Genomes_30x
		South Asian	C=0.1514	G=0.8486	1202	1000Genomes_30x
		Maya	C=0.5	G=0.5	2	Simons Genome Diversity Project
		Mixe	C=0.5	G=0.5	3	Simons Genome Diversity Project
		Pima	C=0.75	G=0.25	2	Simons Genome Diversity Project
		Mixtec	C=0.5	G=0.5	2	Simons Genome Diversity Project
		Zapotec	C=0.75	G=0.25	2	Simons Genome Diversity Project
		Quechua	C=0.833333	G=0.166667	3	Simons Genome Diversity Project
		Surui	C=0	G=1	2	Simons Genome Diversity Project
		Piapoco	C=0	G=1	2	Simons Genome Diversity Project
		Karitiana	C=0.166667	G=0.833333	3	Simons Genome Diversity Project
		Papuan	C=0.2	G=0.8	15	Simons Genome Diversity Project
		Oceania	C=0.16	G=0.84	25	Simons Genome Diversity Project
		Native American	C=0.2365	G=0.7635	1260	The PAGE Study
		Latin American 1	C=1	G=0	2	Allele Frequency Aggregator
		Latin American 2	C=1	G=0	48	Allele Frequency Aggregator
		Native Hawaiian	C=0.1264	G=0.8736	4534	The PAGE Study
	rs1799946	Global	C=0.576078	T=0.423922	264690	TopMed
		American	C=0.696	T=0.304	694	1000Genomes_30x
		African	C=0.4054	T=0.5946	1322	1000Genomes_30x
		European	C=0.6453	T=0.3547	1006	1000Genomes_30x
		East Asian	C=0.5214	T=0.4786	1008	1000Genomes_30x
		South Asian	C=0.5899	T=0.4101	978	1000Genomes_30x
		Maya	C=1	T=0	2	Simons Genome Diversity Project
		Mixe	C=0.833333	T=0.166667	3	Simons Genome Diversity Project
		Pima	C=0.75	T=0.25	2	Simons Genome Diversity Project
		Mixtec	C=0.75	T=0.25	2	Simons Genome Diversity Project
		Zapotec	C=0.75	T=0.25	2	Simons Genome Diversity Project
		Quechua	C=0.833333	T=0.166667	3	Simons Genome Diversity Project
		Surui	C=1	T=0	2	Simons Genome Diversity Project
		Piapoco	C=0.75	T=0.25	2	Simons Genome Diversity Project
		Karitiana	C=1	T=0	3	Simons Genome Diversity Project
		Papuan	C=0.2	T=0.8	15	Simons Genome Diversity Project
		Oceania	C=0.28	T=0.72	25	Simons Genome Diversity Project
		Native American	C=0.6294	T=0.3706	1260	The PAGE Study
		Latin American 1	C=0.551	T=0.449	926	Allele Frequency Aggregator

Gene	rs	Population	Ref	ALT	Sample size	Reference
		Latin American 2	C=0.7068	T=0.2932	2316	Allele Frequency Aggregator
		Native Hawaiian	C=0.6308	T=0.3692	4534	The PAGE Study
	rs11362	Global	C=0.612834	T=0.387166	264690	TopMed
		American	C=0.572	T=0.428	980	1000Genomes_30x
		African	C=0.7066	T=0.2934	1786	1000Genomes_30x
		European	C=0.5616	T=0.4384	1266	1000Genomes_30x
		East Asian	C=0.5744	T=0.4256	1170	1000Genomes_30x
		South Asian	C=0.5616	T=0.4384	1202	1000Genomes_30x
		Maya	C=0.5	T=0.5	2	Simons Genome Diversity Project
		Mixe	C=0.666667	T=0.333333	3	Simons Genome Diversity Project
		Pima	C=1	T=0	2	Simons Genome Diversity Project
		Mixtec	C=0.75	T=0.25	2	Simons Genome Diversity Project
		Zapotec	C=1	T=0	2	Simons Genome Diversity Project
		Quechua	C=1	T=0	3	Simons Genome Diversity Project
		Surui	C=0	T=1	2	Simons Genome Diversity Project
		Piapoco	C=0.25	T=0.75	2	Simons Genome Diversity Project
		Karitiana	C=0.166667	T=0.833333	3	Simons Genome Diversity Project
		Papuan	C=1	T=0	15	Simons Genome Diversity Project
		Oceania	C=0.92	T=0.08	72	HGDP-CEPH-db Supplement 1
		Latin American 1	C=0.632	T=0.368	748	Allele Frequency Aggregator
		Latin American 2	C=0.6023	T=0.3977	6498	Allele Frequency Aggregator
	rs1047031	Global	C=0.859606	T=0.140394	264690	TopMed
		American	C=0.747	T=0.253	980	1000Genomes_30x
		African	C=0.9899	T=0.0101	1786	1000Genomes_30x
		European	C=0.8404	T=0.1596	1266	1000Genomes_30x
		East Asian	C=0.5940	T=0.4060	1170	1000Genomes_30x
		South Asian	C=0.7787	T=0.2213	1202	1000Genomes_30x
		Maya	C=0.75	T=0.25	2	Simons Genome Diversity Project
		Mixe	C=0.666667	T=0.333333	3	Simons Genome Diversity Project
		Pima	C=1	T=0	2	Simons Genome Diversity Project
		Mixtec	C=0.75	T=0.25	2	Simons Genome Diversity Project
		Zapotec	C=1	T=0	2	Simons Genome Diversity Project
		Quechua	C=1	T=0	3	Simons Genome Diversity Project
		Surui	C=0	T=1	2	Simons Genome Diversity Project
		Piapoco	C=0.75	T=0.25	2	Simons Genome Diversity Project
		Karitiana	C=0.666667	T=0.333333	3	Simons Genome Diversity Project
		Papuan	C=1	T=0	15	Simons Genome Diversity Project
		Oceania	C=0.9	T=0.1	25	Simons Genome Diversity Project
		Latin American 1	C=0.8662	T=0.1338	1106	Allele Frequency Aggregator
		Latin American 2	C=0.7640	T=0.2360	6340	Allele Frequency Aggregator
MUC5B	rs2735733	Global	C=0.569111	T=0.430889	264690	TopMed
		American	C=0.453	T=0.547	980	1000Genomes_30x
		African	C=0.7083	T=0.2917	1786	1000Genomes_30x
		European	C=0.5742	T=0.4258	1266	1000Genomes_30x
		East Asian	C=0.3342	T=0.6658	1170	1000Genomes_30x
		South Asian	C=0.5516	T=0.4484	1202	1000Genomes_30x
		Maya	C=0	T=1	2	Simons Genome Diversity Project
		Mixe	C=0	T=1	3	Simons Genome Diversity Project
		Pima	C=0.25	T=0.75	2	Simons Genome Diversity Project
		Mixtec	C=0.25	T=0.75	2	Simons Genome Diversity Project

Gene	rs	Population	Ref	ALT	Sample size	Reference
		Zapotec	C=0.25	T=0.75	2	Simons Genome Diversity Project
		Quechua	C=0.166667	T=0.833333	3	Simons Genome Diversity Project
		Surui	C=0.25	T=0.75	2	Simons Genome Diversity Project
		Piapoco	C=0.5	T=0.5	2	Simons Genome Diversity Project
		Karitiana	C=0.5	T=0.5	3	Simons Genome Diversity Project
		Papuan	C=0.0666667	T=0.933333	15	Simons Genome Diversity Project
		Oceania	C=0.22	T=0.78	25	Simons Genome Diversity Project
		Latin American 1	C=0.494	T=0.506	168	Allele Frequency Aggregator
		Latin American 2	C=0.439	T=0.561	670	Allele Frequency Aggregator
	rs2249073	Global	T=0.475843	C=0.524157	264690	TopMed
		American	T=0.403	C=0.597	980	1000Genomes_30x
		African	T=0.5297	C=0.4703	1786	1000Genomes_30x
		European	T=0.5111	C=0.4889	1266	1000Genomes_30x
		East Asian	T=0.3342	C=0.6658	1170	1000Genomes_30x
		South Asian	T=0.4725	C=0.5275	1202	1000Genomes_30x
		Maya	T=0	C=1	2	Simons Genome Diversity Project
		Mixe	T=0	C=1	3	Simons Genome Diversity Project
		Pima	T=0.25	C=0.75	2	Simons Genome Diversity Project
		Mixtec	T=0.25	C=0.75	2	Simons Genome Diversity Project
		Zapotec	T=0	C=1	2	Simons Genome Diversity Project
		Quechua	T=0.166667	C=0.833333	3	Simons Genome Diversity Project
		Surui	T=0.25	C=0.75	2	Simons Genome Diversity Project
		Piapoco	T=0.5	C=0.5	2	Simons Genome Diversity Project
		Karitiana	T=0.5	C=0.5	3	Simons Genome Diversity Project
		Papuan	T=0.0666667	C=0.933333	15	Simons Genome Diversity Project
		Oceania	T=0.24	C=0.76	25	Simons Genome Diversity Project
		Latin American 1	T=0.432	C=0.568	146	Allele Frequency Aggregator
		Latin American 2	T=0.405	C=0.595	610	Allele Frequency Aggregator
	rs2857476	Global	T=0.453247	C=0.546753	264690	TopMed
		American	T=0.389	C=0.611	980	1000Genomes_30x
		African	T=0.4306	C=0.5694	1786	1000Genomes_30x
		European	T=0.5071	C=0.4929	1266	1000Genomes_30x
		East Asian	T=0.3239	C=0.6761	1170	1000Genomes_30x
		South Asian	T=0.4659	C=0.5341	1202	1000Genomes_30x
		Maya	T=0	C=1	2	Simons Genome Diversity Project
		Mixe	T=0	C=1	3	Simons Genome Diversity Project
		Pima	T=0.25	C=0.75	2	Simons Genome Diversity Project
		Mixtec	T=0.25	C=0.75	2	Simons Genome Diversity Project
		Zapotec	T=0	C=1	2	Simons Genome Diversity Project
		Quechua	T=0.166667	C=0.833333	3	Simons Genome Diversity Project
		Surui	T=0.25	C=0.75	2	Simons Genome Diversity Project
		Piapoco	T=0.5	C=0.5	2	Simons Genome Diversity Project
		Karitiana	T=0.5	C=0.5	3	Simons Genome Diversity Project
		Papuan	T=0.0666667	C=0.933333	15	Simons Genome Diversity Project
		Oceania	T=0.24	C=0.76	25	Simons Genome Diversity Project
		Native American	T=0.4500	C=0.5500	1260	The PAGE Study
		Latin American 1	T=0.458	C=0.542	842	Allele Frequency Aggregator
		Latin American 2	T=0.3901	C=0.6099	6888	Allele Frequency Aggregator
		Native Hawaiian	T=0.4656	C=0.5344	4534	The PAGE Study
		Mexican	T=0.38344	C=0.61656	10810	The PAGE Study

Gene	rs	Population	Ref	ALT	Sample size	Reference
		SouthAmerican	T=0.4072	C=0.5928	1982	The PAGE Study
LTF	rs1126477	Global	C=0.581367	T=0.418633	264690	TopMed
		American	C=0.574	T=0.426	980	1000Genomes_30x
		African	C=0.2228	T=0.7772	1786	1000Genomes_30x
		European	C=0.7322	T=0.2678	1266	1000Genomes_30x
		East Asian	C=0.5812	T=0.4188	1170	1000Genomes_30x
		South Asian	C=0.6223	T=0.3777	1202	1000Genomes_30x
		Maya	C=0.5	T=0.5	2	Simons Genome Diversity Project
		Mixe	C=0.666667	T=0.333333	3	Simons Genome Diversity Project
		Pima	C=0.75	T=0.25	2	Simons Genome Diversity Project
		Mixtec	C=0.25	T=0.75	2	Simons Genome Diversity Project
		Zapotec	C=0.5	T=0.5	2	Simons Genome Diversity Project
		Quechua	C=0.666667	T=0.333333	3	Simons Genome Diversity Project
		Surui	C=0	T=1	2	Simons Genome Diversity Project
		Piapoco	C=0	T=1	2	Simons Genome Diversity Project
		Karitiana	C=0.166667	T=0.833333	3	Simons Genome Diversity Project
		Papuan	C=0.566667	T=0.433333	15	Simons Genome Diversity Project
		Oceania	0.54	T=0.46	25	Simons Genome Diversity Project
		Native American	C=0.6701	T=0.3299	1258	The PAGE Study
		Latin American 1	C=0.5749	T=0.4251	1042	Allele Frequency Aggregator
		Latin American 2	C=0.6313	T=0.3687	6602	Allele Frequency Aggregator
		SouthAmerican	C=0.5822	T=0.4178	1982	The PAGE Study
		Native Hawaiian	C=0.4363	T=0.5637	4534	The PAGE Study
		Mexican	C=0.64150	T=0.35850	10806	The PAGE Study
	rs1126478	Global	T=0.474956	C=0.525044	264690	TopMed
		American	T=0.509	C=0.491	980	1000Genomes_30x
		African	T=0.0364	C=0.9636	1786	1000Genomes_30x
		European	T=0.6493	C=0.3507	1266	1000Genomes_30x
		East Asian	T=0.3410	C=0.6590	1170	1000Genomes_30x
		South Asian	T=0.4551	C=0.5449	1202	1000Genomes_30x
		Maya	T=0.5	C=0.5	2	Simons Genome Diversity Project
		Mixe	T=0.666667	C=0.333333	3	Simons Genome Diversity Project
		Pima	T=0.75	C=0.25	2	Simons Genome Diversity Project
		Mixtec	T=0	C=1	2	Simons Genome Diversity Project
		Zapotec	T=0.5	C=0.5	2	Simons Genome Diversity Project
		Quechua	T=0.666667	C=0.333333	3	Simons Genome Diversity Project
		Surui	T=0	C=1	2	Simons Genome Diversity Project
		Piapoco	T=0	C=1	2	Simons Genome Diversity Project
		Karitiana	T=0.166667	C=0.833333	3	Simons Genome Diversity Project
		Papuan	T=0.433333	C=0.566667	15	Simons Genome Diversity Project
		Oceania	T=0.38	C=0.62	25	Simons Genome Diversity Project
		NativeAmerican	T=0.5825	C=0.4175	1260	The PAGE Study
		Latin American 1	T=0.4632	C=0.5368	1630	Allele Frequency Aggregator
		Latin American 2	T=0.5776	C=0.4224	9350	Allele Frequency Aggregator
		Mexican	T=0.59621	C=0.40379	10810	The PAGE Study
		SouthAmerican	T=0.5303	C=0.4697	1982	The PAGE Study
		Native Hawaiian	T=0.3240	C=0.6760	4534	The PAGE Study
	rs6441989	Global	A=0.451445	G=0.548555	264690	TopMed
		American	A=0.348	G=0.652	980	1000Genomes_30x
		African	A=0.4485	G=0.5515	1786	1000Genomes_30x

Gene	rs	Population	Ref	ALT	Sample size	Reference
		European	A=0.5197	G=0.4803	1266	1000Genomes_30x
		East Asian	A=0.2906	G=0.7094	1170	1000Genomes_30x
		South Asian	A=0.5532	G=0.4468	1202	1000Genomes_30x
		Maya	A=0.5	G=0.5	2	Simons Genome Diversity Project
		Mixe	A=0	G=1	3	Simons Genome Diversity Project
		Pima	A=0	G=1	2	Simons Genome Diversity Project
		Mixtec	A=0.25	G=0.75	2	Simons Genome Diversity Project
		Zapotec	A=0.25	G=0.75	2	Simons Genome Diversity Project
		Quechua	A=0	G=1	3	Simons Genome Diversity Project
		Surui	A=0.75	G=0.25	2	Simons Genome Diversity Project
		Piapoco	A=0.25	G=0.75	2	Simons Genome Diversity Project
		Karitiana	A=0	G=1	3	Simons Genome Diversity Project
		Papuan	A=0	G=1	15	Simons Genome Diversity Project
		Oceania	A=0.12	G=0.88	25	Simons Genome Diversity Project
		Latin American 1	A=0.441	C=0.000, G=0.559	286	Allele Frequency Aggregator
		Latin American 2	A=0.3048	C=0.0000, G=0.6952	1890	Allele Frequency Aggregator
IL-10	rs1800896	American	T=0.691	C=0.309	980	1000Genomes_30x
		African	T=0.6814	C=0.3186	1786	1000Genomes_30x
		European	T=0.5474	C=0.4526	1266	1000Genomes_30x
		East Asian	T=0.9436	C=0.0564	1170	1000Genomes_30x
		South Asian	T=0.7571	C=0.2429	1202	1000Genomes_30x
		Maya	T=0.75	C=0.25	2	Simons Genome Diversity Project
		Mixe	T=1	C=0	3	Simons Genome Diversity Project
		Pima	T=1	C=0	2	Simons Genome Diversity Project
		Mixtec	T=0.75	C=0.25	2	Simons Genome Diversity Project
		Zapotec	T=0.75	C=0.25	2	Simons Genome Diversity Project
		Quechua	T=0.5	C=0.5	3	Simons Genome Diversity Project
		Surui	T=0.75	C=0.25	2	Simons Genome Diversity Project
		Piapoco	T=0.5	C=0.5	2	Simons Genome Diversity Project
		Karitiana	T=0.833333	C=0.166667	3	Simons Genome Diversity Project
		Papuan	T=0.833333	C=0.166667	15	Simons Genome Diversity Project
		Oceania	T=0.833333	C=0.1	25	Simons Genome Diversity Project
		Native American	T=0.6056	C=0.3944	1260	The PAGE Study
		Latin American 1	T=0.6434	C=0.3566	1130	Allele Frequency Aggregator
		Latin American 2	T=0.7164	C=0.2836	7210	Allele Frequency Aggregator
		Mexican	T=0.70370	C=0.29630	10810	The PAGE Study
		SouthAmerican	T=0.7250	C=0.2750	1982	The PAGE Study
		Native Hawaiian	T=0.8013	C=0.1987	4534	The PAGE Study
	rs1800871	Global	A=0.325736	G=0.674264	264690	TopMed
		American	A=0.321	G=0.679	980	1000Genomes_30x
		African	A=0.4362	G=0.5638	1786	1000Genomes_30x
		European	A=0.2417	G=0.7583	1266	1000Genomes_30x
		East Asian	A=0.6726	G=0.3274	1170	1000Genomes_30x
		South Asian	A=0.4609	G=0.5391	1202	1000Genomes_30x
		Maya	A=0.5	G=0.5	2	Simons Genome Diversity Project
		Mixe	A=0.5	G=0.5	3	Simons Genome Diversity Project
		Pima	A=0.5	G=0.5	2	Simons Genome Diversity Project
		Mixtec	A=0.5	G=0.5	2	Simons Genome Diversity Project
		Zapotec	A=0.75	G=0.25	2	Simons Genome Diversity Project

Gene	rs	Population	Ref	ALT	Sample size	Reference
		Quechua	A=0.333333	G=0.666667	3	Simons Genome Diversity Project
		Surui	A=0.5	G=0.5	2	Simons Genome Diversity Project
		Piapoco	A=0	G=1	2	Simons Genome Diversity Project
		Karitiana	A=0.333333	G=0.666667	3	Simons Genome Diversity Project
		Papuan	A=0.2	G=0.8	15	Simons Genome Diversity Project
		Oceania	A=0.34	G=0.66	25	Simons Genome Diversity Project
		Native American	A=0.3079	G=0.6921	1260	The PAGE Study
		Latin American 1	A=0.2986	G=0.7014	1028	Allele Frequency Aggregator
		Latin American 2	A=0.3749	G=0.6251	6588	Allele Frequency Aggregator
		Mexican	A=0.38553	G=0.61447	10806	The PAGE Study
		SouthAmerican	A=0.3607	G=0.6393	1982	The PAGE Study
		Native Hawaiian	A=0.5712	G=0.4288	4534	The PAGE Study
	rs1800872	Global	T=0.325751	G=0.674249	264690	TopMed
		American	T=0.321	G=0.679	980	1000Genomes_30x
		African	T=0.4362	G=0.5638	1786	1000Genomes_30x
		European	T=0.2417	G=0.7583	1266	1000Genomes_30x
		East Asian	T=0.6726	G=0.3274	1170	1000Genomes_30x
		South Asian	T=0.4617	G=0.5383	1202	1000Genomes_30x
		Maya	T=0.5	G=0.5	2	Simons Genome Diversity Project
		Mixe	T=0.5	G=0.5	3	Simons Genome Diversity Project
		Pima	T=0.5	G=0.5	2	Simons Genome Diversity Project
		Mixtec	T=0.5	G=0.5	2	Simons Genome Diversity Project
		Zapotec	T=0.75	G=0.25	2	Simons Genome Diversity Project
		Quechua	T=0.333333	G=0.666667	3	Simons Genome Diversity Project
		Surui	T=0.5	G=0.5	2	Simons Genome Diversity Project
		Piapoco	T=0	G=1	2	Simons Genome Diversity Project
		Karitiana	T=0.333333	G=0.666667	3	Simons Genome Diversity Project
		Papuan	T=0.2	G=0.8	15	Simons Genome Diversity Project
		Oceania	T=0.34	G=0.66	25	Simons Genome Diversity Project
		Latin American 1	T=0.280	G=0.720	168	Allele Frequency Aggregator
		Latin American 2	T=0.361	G=0.639	670	Allele Frequency Aggregator
CRACR2A	rs242016	Global	G=0.878756	A=0.121244	264690	TopMed
		American	G=0.890	A=0.110	980	1000Genomes_30x
		African	G=0.9580	A=0.0420	1786	1000Genomes_30x
		European	G=0.8483	A=0.1517	1266	1000Genomes_30x
		East Asian	G=0.9957	A=0.0043	1170	1000Genomes_30x
		South Asian	G=0.9376	A=0.0624	1202	1000Genomes_30x
		Maya	G=1	A=0	2	Simons Genome Diversity Project
		Mixe	G=1	A=0	3	Simons Genome Diversity Project
		Pima	G=1	A=0	2	Simons Genome Diversity Project
		Mixtec	G=1	A=0	2	Simons Genome Diversity Project
		Zapotec	G=1	A=0	2	Simons Genome Diversity Project
		Quechua	G=1	A=0	3	Simons Genome Diversity Project
		Surui	G=1	A=0	2	Simons Genome Diversity Project
		Piapoco	G=1	A=0	2	Simons Genome Diversity Project
		Karitiana	G=1	A=0	3	Simons Genome Diversity Project
		Papuan	G=1	A=0	15	Simons Genome Diversity Project
		Oceania	G=1	A=0	25	Simons Genome Diversity Project
		Latin American 1	G=0.882	A=0.118	654	Allele Frequency Aggregator

Gene	rs	Population	Ref	ALT	Sample size	Reference
		Latin American 2	G=0.9241	A=0.0759	2834	Allele Frequency Aggregator

Ref = Reference allele. As stated by NCBI dbSNP Short Genetic Variations

ALT= Alternative allele. As stated by NCBI dbSNP Short Genetic Variations

Rs= Reference SNP cluster ID

Internet Resources

1000 Genomes Project (2023) Genome, https://www.genome.gov/27528684/1000-genomes-project (accessed 23 April 2023).

Allele Frequency Aggregator (2023) ALFA: Allele Frequency Aggregator, https://www.ncbi.nlm.nih.gov/snp/docs/gsr/alfa/ (accessed 23 April 2023).

Simons Genome Diversity Project (2023) https://www.simonsfoundation.org/simons-genome-diversity-project/ (accessed 23 April 2023).

The PAGE Study (2023) Population Architecture using Genomics and Epidemiology, https://www.pagestudy.org/ (accessed 20 April 2023).

TOPMed (2023) NHLBI TOPMed: Phase III variation data, https://www.ncbi.nlm.nih.gov/bioproject/PRJNA400167 (accessed 23 April 2023).
