# Supplementary material for: Oral microbiota, co-evolution, and implications for health and disease: The case of indigenous peoples
Source: Genet Mol Biol. 2024 Jan 22;46(3 Suppl 1):e20230129. doi: 10.1590/1678-4685-GMB-2023-0129 (PMC10829892; doi:10.1590/1678-4685-GMB-2023-0129)
Supplement: Table S5 - [file 1415-4757-GMB-46-03-s1-e20230129-s5.pdf]

Genetics and Molecular Biology

https://doi.org/10.1590/1678-4685-GMB-2023-0129

Supplementary Material to "Oral microbiota, co-evolution, and implications for health and disease: the case of indigenous peoples"

Table S5 - χ^2 statistical test calculated for African, European, East Asian, South Asian, Native American, and Native Hawaiian major human groups based on allele frequencies‡.

Genes	rs	χ^2	p-value	Degree of freedom
DEFB1	rs1800972	611.3534	7.12319646741302e-130	df = 5
	rs1799946	514.0647	7.34745225378565e-109	df = 5
	rs11362	201.4466	2.05387713113226e-43	df = 3
	rs1047031	1561.9439	0	df = 3
MUC5B	rs2735733	806.3139	1.84878281687632e-174	df = 3
	rs2249073	241.4913	4.52888586312589e-52	df = 3
	rs2857476	200.2945	2.45700159005223e-41	df = 5
LTF	rs1126477	2287.1235	0	df = 5
	rs1126478	3234.7555	0	df = 5
	rs6441989	392.1157	1.12950340272848e-84	df = 3
IL-10	rs1800896	1479.2533	9.22538148409318e-318	df = 5
	rs1800871	1555.6394	0	df = 5
	rs1800872	917.3240	1.54630151443081e-198	df = 3
CRACR2A	rs242016	493.2939	1.35502056785091e-106	df = 3

‡Table S4.
