# Supplementary material for: Oral microbiota, co-evolution, and implications for health and disease: The case of indigenous peoples
Source: Genet Mol Biol. 2024 Jan 22;46(3 Suppl 1):e20230129. doi: 10.1590/1678-4685-GMB-2023-0129 (PMC10829892; doi:10.1590/1678-4685-GMB-2023-0129)
Supplement: Table S6 - [file 1415-4757-GMB-46-03-s1-e20230129-s6.pdf]

*Genetics and Molecular Biology*

https://doi.org/10.1590/1678-4685-GMB-2023-0129

# Supplementary Material to "Oral microbiota, co-evolution, and implications for health and disease: the case of indigenous peoples"

**Table S6 -** Bacterial species analyzed for the *16S rRNA* gene and their respective references.

| Access number | Species |
|---|---|
| LN624398.1 | *Actinomyces gerencseriae* |
| 618_0414 | *Actinomyces gerencseriae* |
| NR_026227.1 | *Actinomyces israelii* |
| 645_2450 | *Actinomyces israelii* |
| AB618790.1 | *Actinomyces naeslundii* |
| 176_1062 | *Actinomyces naeslundii* |
| NR_104896.1 | *Actinomyces oris* |
| 893_5935 | *Actinomyces oris* |
| FN547968.1 | *Aggregatibacter actinomycetemcomitans* |
| 531_5035 | *Aggregatibacter actinomycetemcomitans* |
| NR_043605.1 | *Campylobacter gracilis* |
| 623_4320 | *Campylobacter gracilis* |
| NR_043606.1 | *Campylobacter rectus* |
| 748_4317 | *Campylobacter rectus* |
| NR_043601.1 | *Campylobacter showae* |
| 763_6974 | *Campylobacter showae* |
| NR_026094.1 | *Capnocytophaga gingivalis* |
| 337_7608 | *Capnocytophaga gingivalis* |
| NR_027581.1 | *Capnocytophaga ochracea* |
| 700_4635 | *Capnocytophaga ochracea* |
| NR_026095.1 | *Capnocytophaga sputigena* |
| 775_7609 | *Capnocytophaga sputigena* |
| 530_5256 | *Cutibacterium acnes* |
| DQ672261.1 | *Cutibacterium acnes* |
| NR_104685.1 | *Eikenella corrodens* |
| 577_0620 | *Eikenella corrodens* |
| NR_118781.1 | *Eubacterium nodatum* |
| 694_6274 | *Eubacterium nodatum* |
| NR_026083.1 | *Fusobacterium nucleatum.* |
| 698_3496 | *Fusobacterium nucleatum* |
| 201_5405 | *Fusobacterium periodonticum* |
| NR_026085.1 | *Fusobacterium periodonticum* |
| LC096237.1 | *Gemella morbillorum* |
| 046_4327 | *Gemella morbillorum* |
| NR_104684.1 | *Lachnoanaerobaculum saburreum* |
| 494_RDP | *Lachnoanaerobaculum saburreum* |

| **Access number** | **Species** |
| --- | --- |
| NR_074440.1 | *Leptotrichia buccalis* |
| 563_7788 | *Leptotrichia buccalis* |
| NR_117696.1 | *Neisseria mucosa* |
| 682_9282 | *Neisseria mucosa* |
| D14143.1 | *Parvimonas micros* |
| 111_3523 | *Parvimonas micra* |
| NR_040838.1 | *Porphyromonas gingivalis* |
| 619_3964 | *Porphyromonas gingivalis* |
| NR_026119.1 | *Prevotella intermedia* |
| 643_2907 | *Prevotella intermedia* |
| NR_042843.1 | *Prevotella melaninogenica* |
| 469_3525 | *Prevotella melaninogenica* |
| NR_044850.1 | *Prevotella nigrescens* |
| 693_3963 | *Prevotella nigrescens* |
| AJ234040.1 | *Schaalia odontolyticus* |
| 701_0504 | *Schaalia odontolyticus* |
| 130Snoxi | *Selenomonas noxia* |
| LC037221.1 | *Selenomonas noxiar* |
| NR_041722.2 | *Streptococcus anginosus* |
| 543_4678 | *Streptococcus anginosus* |
| NR_041721.1 | *Streptococcus constellatus* |
| 576_4676 | *Streptococcus constellatus* |
| NR_028666.1 | *Streptococcus gordonii* |
| 622_3931 | *Streptococcus gordonii* |
| NR_028736.1 | *Streptococcus intermedius* |
| 644_4671 | *Streptococcus intermedius* |
| NR_028664.1 | *Streptococcus mitis* |
| 677_3929 | *Streptococcus mitis* |
| NR_042772.1 | *Streptococcus mutans* |
| 686_3965 | *Streptococcus mutans* |
| NR_042927.1 | *Streptococcus oralis* |
| 707_3932 | *Streptococcus oralis* |
| NR_024841.1 | *Streptococcus sanguinis* |
| 758_3928 | *Streptococcus sanguinis* |
| NR_040839.1 | *Tannerella forsythia* |
| 613_6495 | *Tannerella forsythia* |
| NR_074582.1 | *Treponema denticola* |
| 584_1236 | *Treponema denticola* |
| NR_028690.1 | *Treponema socranskii* |
| 769_3307 | *Treponema socranskii* |
| NR_043332.1 | *Veillonella parvula* |
| 161Vparv | *Veillonella parvula* |
| FR870448.1 | *Halorubrum tebenquich* |
| X90482.1 | *Metallosphaera prunae* |
| AJ318041.1 | *Nanoarchaeum equitans* |
