# Supplementary material for: Oral microbiota, co-evolution, and implications for health and disease: The case of indigenous peoples
Source: Genet Mol Biol. 2024 Jan 22;46(3 Suppl 1):e20230129. doi: 10.1590/1678-4685-GMB-2023-0129 (PMC10829892; doi:10.1590/1678-4685-GMB-2023-0129)
Supplement: Table S7 - [file 1415-4757-GMB-46-03-s1-e20230129-s7.pdf]

Genetics and Molecular Biology

https://doi.org/10.1590/1678-4685-GMB-2023-0129

Supplementary Material to "Oral microbiota, co-evolution, and implications for health and disease: the case of indigenous peoples"

Table S7 - Microbial composition of the supra and subgingival plaque classified by complex. NI are species not considered by the authors, while "other species" includes those that did not fit into the complexes.

**Bacterial species**	**Socransky *et al.* (1998)**	**Ximénez-Fyvie *et al.* (2000)**	**Socransky *et al.* (2002)**	**Socransky and Haffajee (2002)**	**Uzel *et al.* (2011)**
Aggregatibacter actinomycetemcomitans B	Purple	NI	Other species	NI	NI
Campylobacter concisus	Green	NI	Green	NI	NI
Streptococcus gordonii	Yellow	Yellow	Yellow	Yellow	Yellow
Streptococcus intermedius	Yellow	Yellow	Yellow	Yellow	Yellow
Streptococcus mitis	Yellow	Yellow	Yellow	Yellow	Yellow
Streptococcus oralis	Yellow	Yellow	Yellow	Yellow	Yellow
Streptococcus sanguinis	Orange	Yellow	Yellow	Yellow	Yellow
Actinomyces naeslundii	Purple	*Actinomyces* group	*Actinomyces* group	Blue	Blue
Actinomyces gerencseriae	NI	*Actinomyces* group	NI	Blue	Blue
Actinomyces israelii	NI	*Actinomyces* group	NI	Blue	Blue
Actinomyces oris	NI	NI	NI	NI	Blue
Selenomonas noxiar	Purple	Other species	Other species	Other species	Other species
Streptococcus constellatus	Orange	Orange	Orange	Orange	Orange
Eubacterium nodatum	Orange	Orange	Orange	Orange	Orange
Peptostreptococcus micros	Orange	Orange	Orange	Orange	Orange
Fusobacterium nucleatum.	Orange	Orange	Orange	Orange	Orange
Fusobacterium periodonticum	Orange	Orange	Orange	Orange	Orange
Campylobacter gracilis	Orange	Orange	Orange	Orange	Orange
Campylobacter rectus	Orange	Orange	Orange	Orange	Orange
Campylobacter showae	Orange	Orange	Orange	Orange	Orange
Prevotella intermedia	Orange	Orange	Orange	Orange	Orange
Prevotella nigrescens	Orange	Orange	Orange	Orange	Orange
Veillonella parvula	Purple	Purple	Purple	Purple	Purple
Actinomyces odontolyticus	Purple	Purple	Purple	Purple	Purple
Aggregatibacter actinomycetemcomitans A	Green	Green	Green	Green	Green
Eikenella corrodens	Green	Green	Green	Green	Green
Capnocytophaga ochracea	Green	Green	Green	Green	Green
Capnocytophaga sputigena	Green	Green	Green	Green	Green
Capnocytophaga gingivalis	Green	Green	Green	Green	Green

**Bacterial species**	**Socransky *et al.* (1998)**	**Ximénez-Fyvie *et al.* (2000)**	**Socransky *et al.* (2002)**	**Socransky and Haffajee (2002)**	**Uzel *et al.* (2011)**
Treponema denticola	Red	Red	Red	Red	Red
Porphyromonas gingivalis	Red	Red	Red	Red	Red
Tannerella forsythia	Red	Red	Red	Red	Red
Eubacterium saburreum	NI	Other species	NI	NI	Other species
Gemella morbillorum	NI	Other species	NI	Other species	Other species
Leptotrichia buccalis	NI	Other species	NI	Other species	Other species
Neisseria mucosa	NI	Other species	NI	Other species	Other species
Prevotella melaninogenica	NI	Other species	NI	Other species	Other species
Propionibacterium acnes	NI	Other species	NI	Other species	Other species
Streptococcus anginosus	NI	Other species	NI	Other species	Other species
Treponema socranskii	NI	Other species	NI	Other species	Other species
Streptococcus mutans	NI	NI	NI	NI	Other species

References

Socransky SS, Haffajee AD, Cugini MA, Smith CKJR and Kent Jr RL (1998) Microbial complexes in subgingival plaque. J Clin Periodontol 25:134-144.

Socransky SS and Haffajee AD (2002) Dental biofilms: Difficult therapeutic targets. Periodontol 2000 28:12-55.

Socransky SS, Smith C and Haffajee AD (2002) Subgingival microbial profiles in refractory periodontal disease. J Clin Periodontol 29:260-268.

Uzel NG, Teles FR, Teles RP, Song XQ, Torresyap G, Socransky SS and Haffajee AD (2011) Microbial shifts during dental biofilm re-development in the absence of oral hygiene in periodontal health and disease. J Clin Periodontol 38:612-620.

Ximénez-Fyvie LA, Haffajee AD and Socransky SS (2000) Comparison of the Microbiota of supra-and subgingival plaque in health and periodontitis. J Clin Periodontol, 27:648-657.
