# Supplementary material for: Oral microbiota, co-evolution, and implications for health and disease: The case of indigenous peoples
Source: Genet Mol Biol. 2024 Jan 22;46(3 Suppl 1):e20230129. doi: 10.1590/1678-4685-GMB-2023-0129 (PMC10829892; doi:10.1590/1678-4685-GMB-2023-0129)
Supplement: Figure S1 - [file 1415-4757-GMB-46-03-s1-e20230129-s8.pdf]

Genetics and Molecular Biology

https://doi.org/10.1590/1678-4685-GMB-2023-0129

Supplementary Material to "Oral microbiota, co-evolution, and implications for health and disease: the case of indigenous peoples"

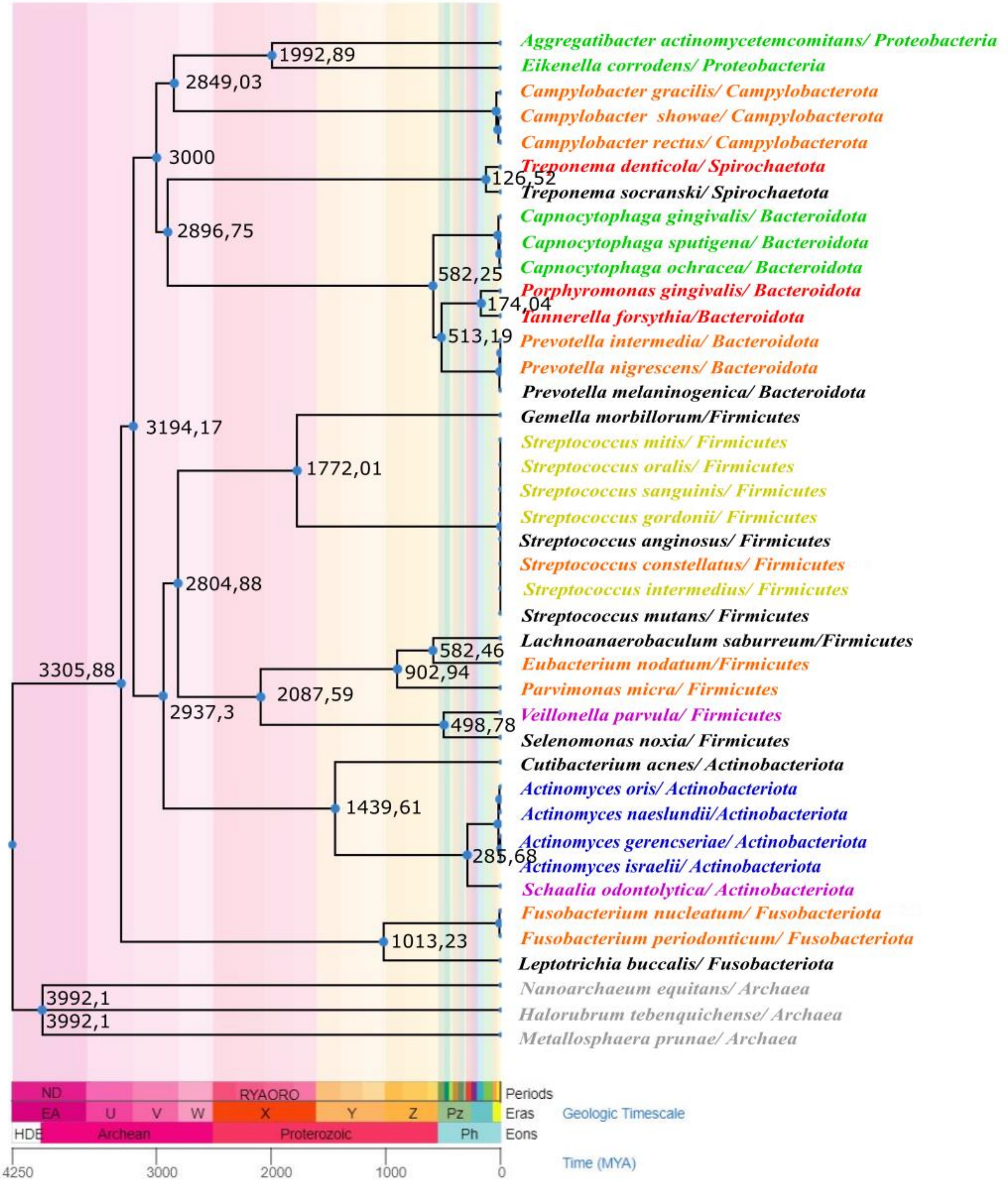

Figure S1 - Divergence time tree considering 39 bacterial taxa described in Uzel et al. (2011). Species are color-coded according to previously described microbial complexes (Socransky et al., 1998). Phylogeny was performed in Timetree. The numbers (years) in the nodes must be multiplied by 1,000. The Phyla nomenclature is according to the GTDB.
