# Supplementary material for: Oral microbiota, co-evolution, and implications for health and disease: The case of indigenous peoples
Source: Genet Mol Biol. 2024 Jan 22;46(3 Suppl 1):e20230129. doi: 10.1590/1678-4685-GMB-2023-0129 (PMC10829892; doi:10.1590/1678-4685-GMB-2023-0129)
Supplement: Figure S2 - [file 1415-4757-GMB-46-03-s1-e20230129-s9.pdf]

Genetics and Molecular Biology

https://doi.org/10.1590/1678-4685-GMB-2023-0129

Supplementary Material to "Oral microbiota, co-evolution, and implications for health and disease: the case of indigenous peoples"

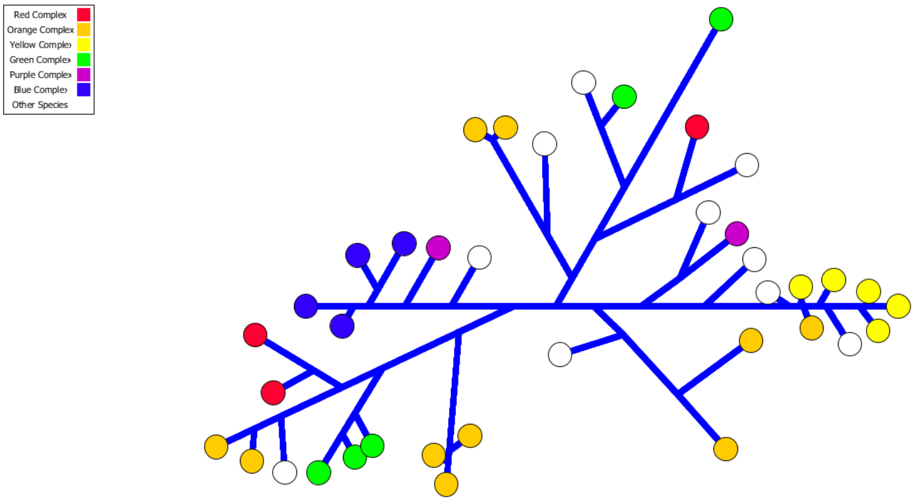

Figure S2 - *16srRNA* sequence-based haplotype network considering 39 bacterial taxa described in Uzel et al. (2011). Species are color-coded according to previously described microbial complexes (Socransky et al., 1998).
