# Supplementary material for: Oral microbiota, co-evolution, and implications for health and disease: The case of indigenous peoples
Source: Genet Mol Biol. 2024 Jan 22;46(3 Suppl 1):e20230129. doi: 10.1590/1678-4685-GMB-2023-0129 (PMC10829892; doi:10.1590/1678-4685-GMB-2023-0129)
Supplement: Supplementary file 10 [file 1415-4757-GMB-46-03-s1-e20230129-s10.pdf]

Genetics and Molecular Biology

https://doi.org/10.1590/1678-4685-GMB-2023-0129

Supplementary Material to "Oral microbiota, co-evolution, and implications for health and disease: the case of indigenous peoples"

<u>Additional information regarding the main topics of the review</u>

Human oral microbiota, its composition, and its complexity: selected information on CPR, archaean, fungi, viruses, and protozoan species

In addition to bacteria, the human oral cavity is also home to ultra-small bacteria belonging to the newly classified group of "candidate phyla radiation" (or CPR; Hug *et al.*, 2000), whose culture *in vitro* has been quite difficult to obtain. Besides, it is unknown how CPR members interact with the host and other agents that make up the oral microbiota. However, it is known three phyla: Gracilibacteria (also known as GN02), Absconditabacteria (SR1), and Saccharibacteria (TM7). CPR Saccharibacteria is known to be associated with multiple mucosal diseases, including periodontitis, highlighting the ability of these ultra-small bacteria to contribute to human disease and modulate the host immune response (He, 2015; Naud *et al.*, 2022).

The first members of the Archaea domain detected in the oral cavity were methanogenic organisms (releasing methane gas as a metabolic waste) recovered from subgingival plaque (Brusa *et al.* 1987). A later study postulated that there was a connection between higher proportions of methanogenic Archaean species and the severity of Periodontal Diseases (PDs) (Belay *et al.*, 1988). Recent investigations have shown that *Methanobrevibacter oralis* is the most abundant oral Archaean species (Belmok *et al.*, 2020). The abundance of *Methanobrevibacter oralis* was correlated with the presence of bacteria *Porphyromonas gingivalis, Tannerella*

forsythia, and/or *Prevotella intermedi* in PD patients (Matarazzo *et al.*, 2012; Horz *et al.*, 2015; Belmok *et al.*, 2020). On the other hand, a high prevalence of *Treponema denticola* (red complex) was found in PD sites (or habitats) without methanogenic Archaea (Lepp *et al.*, 2004). These findings indicate a complex symbiotic relationship between such organisms because mutualism and antagonism seem to exist.

The non-bacterial component of the human microbiota is poorly studied compared to the bacterial element. However, it is known, for example, that a healthy oral cavity harbors more than 75 genera of fungi; *Candida*, *Cladosporium*, *Aureobasidium*, and *Aspergillus,* the latter being the unicellular eukaryote genera most abundant in the oral cavity, found in 25-75% of people investigated (Ghannoum, 2010). More recurrently, the sequence of the internal transcript spacer (ITS), a non-functional segment of rRNA present in fungi, is used to identify members of this type of organism since it has similar characteristics as a phylogenetic marker of the 16SrRNA in bacteria and Archaea.

Identifying the human virome of any organ or tissue, in turn, has been challenging since viruses can have DNA or RNA (double-stranded or single-stranded) as genetic material and an extraordinary variability within and between taxa. Thus, there is a loss of a universal genetic marker for this type of organism. Therefore, large-scale sequencing techniques have revolutionized the identification of viromes. Despite these advances, there are many steps suggested to identify correct viral genomes since some of them, especially from bacteriophages (viruses specialized in infecting bacteria), have a large proportion of "dark matter", *i.e.*, sequences that do not resemble anything previously deposited in the databases (Liang and Bushman, 2021).

Human oral virome studies revealed that there are ~10^8 viral particles per milliliter of saliva, the most frequent being viruses from the families Herpesviridae, Redondoviridae, Anelloviridae, and Papillomaviridae, while bacteriophages are those belonging to three families, Siphoviridae, Podoviridae, and Myoviridae (Abeles *et al.*, 2014; Shkoporov *et al.*, 2018; Liang

and Bushman, 2021). Anelloviridae is the most common, followed by viruses from the newly discovered Redondoviridae family (Mirzaei and Maurice, 2017; Liang and Bushman, 2021). As bacteriophages infect opportunistic pathogenic species, such as those of the *Aggregatibacter* and *Haemophilus* genera, they impact human microbial ecology and pathologies (Szafrański *et al.*, 2019). Members of the three bacteriophage families mentioned above infect all major bacterial phyla found in the oral cavity (Pride *et al.*, 2012). So, it can be assumed that there are reciprocal selective pressures, but they have not been studied to date.

Protozoans also cause diseases in the oral cavity. For example, *Trichomonas tenax* was three times more frequent in the case of severe PD, while *Entamoeba gingivalis* reached its highest frequency in cases of gingivitis (Tan and Li, 2021; Puzio *et al.*, 2021).

Diversity of the oral microbiota in non-human primates

Möller *et al.* (1981) conducted a comparison involving 78 necrotic pulps from nine crab-eating macaques (*Macaca fascicularis*), with 26 pulps being bacteria-free and 52 pulps infected by indigenous oral flora. The study revealed that non-infected necrotic pulp did not trigger inflammatory reactions. However, infected pulps exhibited these reactions both in clinical and radiographic analyses. Furthermore, none of the samples taken for cultivation or microscopy from the non-infected teeth group displayed any presence of bacteria, either in the initial or final samples. In contrast, the most commonly identified microorganisms in both initial and final samples of the infected teeth group included facultatively anaerobic bacteria (*e.g.*, *Streptococcus* ssp., *Enterococcus* ssp., and coliform rods), along with obligatory anaerobic bacteria species (*e.g.*, *Eubacterium* ssp. *Propionibacterium* ssp., *Peptococcus* ssp., and *Peptostreptococcus* ssp.).

Other evolutionary dynamics operating in the microbiota

In addition to the arms race and Red or Black Queen dynamics, the literature offers other intriguing concepts. One such example is the "keystone pathogen hypothesis," introduced by Hajishengallis *et al.* (2012), which proposes that specific microorganisms within a microbial

community can disproportionately influence the structure and function of an ecosystem, similar to a keystone species in an ecological community. This idea underscores the role of certain pathogens in driving dysbiosis and disease progression, even when present in low abundance. For instance, pathogens with low abundance can orchestrate inflammatory diseases. This notion gains support from the observation that *Porphyromonas gingivalis* (part of the red complex) employs sophisticated strategies to evade or manipulate components of the host immune system, rather than being a straightforward pro inflammatory bacterium. Consequently, it is suggested that *P. gingivalis* impairs innate immunity, influencing the overall growth and development of the biofilm and triggering a detrimental shift in the usual interaction between the host and microbial communities in the periodontium. Essentially, *P. gingivalis* could act as a keystone pathogen within the disease-associated periodontal microbiota, transforming a typically benign microbiota into a dysbiotic state. A mouse model can illustrate the keystone pathogen hypothesis. Even at shallow levels of colonization (<0.01% of total bacterial count), *P. gingivalis* triggered periodontitis, causing significant changes in the abundance and disposition of oral commensal bacteria in mice. These changes occurred shortly after colonization by *P. gingivalis*, preceding the onset of inflammatory bone loss, suggesting that dysbiosis is likely behind the cause of the disease. The vital role of the commensal microbiota in disease development was demonstrated when *P. gingivalis* failed to induce periodontitis in germ-free mice, despite its ability to colonize this host (Hajishengallis *et al.*, 2012 and references therein).

Indigenous peoples from other continents and ethical concerns

Although there are many differences between continental native populations, here we focus on the case of Indigenous populations as defined by the United Nations (see main text).

Regarding Indigenous communities, in addition to the Native American cases mentioned in the main text of this review, there are further illustrations worth noting. For instance, Lassalle *et al.* (2018) examined the oral microbiota of three pairs of Filipino Indigenous groups residing in close proximity to each other, sharing the same environment: Batak and Tagbanua, Aeta and

Zambal, and Agta and Casigurani were classified respectively as hunter-gatherers and traditional farmers. The authors point out that there was a higher prevalence of species previously associated with PDs (*Prevotella intermedia, Porphyromonas gingivalis, Treponema denticola, Tannerella forsythia, Aggregatibacter actinomycetemcomitans*, and *Eubacterium nodatum*) among hunter-gatherers than among traditional farmers, although the former presented much better oral health (Lassalle *et al.*, 2018).

Dobon *et al.* (2023) also showed that the oral microbiota of the Agta hunter-gatherers had changed substantially since some of their members started to adopt an agricultural diet rich in rice, concomitant with the abandonment of a more traditional diet rich in fish/meat, mirroring the Neolithic transition. The authors detected that the oral microbiota of Agta with the traditional diet had large amounts of species of the genera *Actinobacillus, Alphaproteobacteria*, and *Streptobacillus* and a lower abundance of species of the genera *Selenomonas, Atopobium, Peptoanaerobacter*, and *Pyramidobacter*. The authors found fascinating that those individuals consuming a diet rich in animal proteins had a greater abundance of *Actinobacillus*, given the extraordinary proteolytic potential of *Aggregatibacter actinomycetencomitans*, an oral pathogen frequently found in aggressive forms of periodontitis. In contrast, the rice-eating Agta showed increased caries-causing bacteria, such as species of the genus *Scardovia* (Dobon *et al.*, 2023). All of these factors have implications for the epidemiology of oral diseases in Agta individuals. The authors also pointed out that there was a straightforward co-evolutionary process between the infectious agents and their host, but identifying markers of this process in the genomes remained enormously challenging (Dobon *et al*., 2023).

Handsley-Davis *et al.* (2022), in turn, performed the first study examining oral microbiota in Indigenous Australian adults, encompassing Aboriginal Australians and Torres Strait Islanders. The researchers compared dental calculus samples from Indigenous individuals with PD, as well as non-Indigenous individuals with and without PD, using *16SrRNA*. Results revealed that samples from Indigenous Australians exhibited higher phylogenetic diversity, significant

compositional differences from non-Indigenous samples, and contained a relatively high proportion of unique taxa not previously found in human oral microbiota (*e.g*., Endomicrobia). Additionally, microbiota diversity and composition varied among Indigenous individuals across geographic regions. Notably, a single feature in the genus *Porphyromonas* was significantly more prevalent in Indigenous Australians than in non-Indigenous Australians. Among Indigenous Australians, 171 unique microbial features were identified in at least two samples. The top species abundances were belonging to the families Mogibacteriaceae, Tissierellaceae, and Desulfomicrobiaceae, as well as genera *Porphyromonas* ssp. and *Methanobrevibacter* ssp. For non-Indigenous Australians, the top features included species from the genera *Streptococcus*, *Rothia*, *Fusobacterium*, and species belonging to the family Peptostreptococcaceae. These findings suggested potential differences in oral microbe strains between Indigenous and non-Indigenous Australians.

Another theme that deserves additional comment concerns the importance of ethical practices in microbiome research, mainly when working with Indigenous peoples (Mangola *et al*., 2022). The last authors discuss the need for inclusive research practices, such as community-based participatory research and culturally appropriate sample collection protocols. The authors also emphasize the importance of long-term partnerships, benefit-sharing agreements, and the use of tiered informed consent. Additionally, they highlight the need for transparency, accountability, and benefit sharing with participant communities in cross-cultural microbiome research. They also stress the importance of capacity building, including Indigenous scholars, and appropriate language and descriptions when discussing communities. The paper calls for further dialogue and collaboration between scientists and participant communities to address these ethical considerations. Lastly, the authors argue for equitable collaboration, power sharing, and the decolonization of research methodologies in anthropology and advocate for the inclusion of marginalized communities in the research process (Mangola *et al*., 2022).

Material and methods

Times of divergence and phylogenetic tree

Based on studies of bacterial complexes in the oral microbiome by Socransky *et al.* (1998) and Uzel *et al.* (2011) we selected 81 *16SrRNA* sequences from available databases (Table S6), the National Center for Biotechnology Information (NCBI) and the Human Oral Microbiome Database (HOMD, 2023), with 39 species described as belonging to the oral microbiome, each species with a duplicate for each database and 3 representative species of the domain Archaea. We used *16SrRNA* sequences because it is considered the primary genetic locus in the classification and characterization of bacterial species due to its universality and nature similar to a molecular clock and because it has multiple conserved/hypervariable regions (Woese, 1987; Van de Peer, 1996).

Sequence alignment was performed in Guidance 2 (Sela *et al.*, 2015), using the MAFFT method. We inferred the Maximum Likelihood tree using the IQ-TREE web server program (Trifinopoulos *et al.*, 2016) with the ideal substitution model GTR+I+G estimated with jModelTest v.2.1.10 program (Darriba *et al.*, 2012) and used in the IQ-TREE under the Akaike Information Criterion (AIC). We performed 10,000 ultrafast bootstrap (BS) replications (Minh *et al.*, 2013) and 1,000,000 maximum iterations. The resulting consensus tree was visualized and edited with FigTree 1.4.4 (Rambaut, 2018). The divergence time tree was reconstructed by the Timetree platform (Kumar *et al.*, 2022).

The DnaSP v.6 program (Rozas *et al.*, 2017) was used to identify haplotypes of 16S rRNA sequences and generate data matrices. The hierarchical relationships between haplotypes were observed in haplotype networks generated by the Haplotype Viewer program (Salzburger *et al.*, 2011), which is based on the implementation of the Phylip package algorithm (phylogeny inference package) that generates a tree from maximum likelihood method (DNAML) (Felsenstein, 1993).

Selected SNPs with PDs and/or caries association

After carefully reviewing the existing literature, we selected a subset of genetic variants that are commonly associated with periodontitis and caries in diverse human populations (Table S3-S4). Our aim was to illustrate the impact of genetic variations within the host's genome on their ability to respond to microbial challenges. We chose 14 SNPs (Single nucleotide polymorphisms) associated with 5 genes and compared their allelic frequencies across various continental groups available in NCBI dbSNP Short Genetic Variations database, as well as among some indigenous populations, using BCF (Li, 2011) and VCF tools (Danecek *et al.*, 2011) (Table S3-S4). Subsequently, we performed a chi-square test to verify if the differences were statistically significant using R environment (R Core Team, 2021).

References

Abeles SR, Robles-Sikisaka R, Ly M, Lum AG, Salzman J, Boehm TK and Pride DT (2014) Human oral viruses are personal, persistent and gender-consistent. ISME J 8:1753-1767.

Belay N, Johnson R, Rajagopal BS, Conway de Macario E and Daniels L (1988) Methanogenic bacteria from human dental plaque. Appl Environ Microbiol 54:600-603.

Belmok A, De Cena JA, Kyaw CM and Damé-Teixeira N (2020) The oral archaeome: A scoping review. J Dent Res 99:630-643.

Brusa T, Conca R, Ferrara A, Ferrari A and Pecchioni A (1987) The presence of methanobacteria in human subgingival plaque. J Clin Periodontol 14:470-471.

Danecek P, Auton A, Abecasis G, Albers CA, Banks E, DePristo MA, Handsaker RE, Lunter G, Marth GT, Sherry ST *et al.* (2011) The variant call format and VCFtools. Bioinformatics 27:2156-2158.

Darriba D, Taboada GL, Doallo R and Posada D (2012) jModelTest 2: More models, new heuristics and parallel computing. Nat Methods 9:772-772.

Dobon B, Musciotto F, Mira A, Greenacre M, Schlaepfer R, Aguileta G, Astete LH, Ngales M, Latora V, Battiston F *et al.* (2023) The making of the oral microbiome in Agta hunter-gatherers. Evol Hum Sci 5:e13.

Felsenstein J (1993) Phylip (phylogeny inference package), version 3.5c. Department of Genetics, University of Washington, Seattle.

Ghannoum MA (2010) Characterization of the oral fungal microbiome (mycobiome) in healthy individuals. PLoS Pathog 6:e1000713.

Hajishengallis G, Darveau RP and Curtis MA (2012) The keystone-pathogen hypothesis. Nat Rev Microbiol 10:717-725.

Handsley-Davis M, Kapellas K, Jamieson LM, Hedges J, Skelly E, Kaidonis J, Anastassiadis P and Weyrich LS (2022) Heritage-specific oral microbiota in Indigenous Australian dental calculus. Evol Med Public Health 10:352-362.

He X (2015) Cultivation of a human-associated TM7 phylotype reveals a reduced genome and epibiotic parasitic lifestyle. Proc Natl Acad Sci U S A 112:244-249.

Horz HP, Robertz N, Vianna ME, Henne K and Conrads G (2015) Relationship between methanogenic archaea and subgingival microbial complexes in human periodontitis. Anaerobe 35:10-12.

Hug LA, Baker BJ, Anantharaman K, Brown CT, Probst AJ, Castelle CJ, Butterfield CN, Hernsdorf AW, Amano Y, Ise K *et al.* (2000) A new view of the tree of life. Nat Microbiol 1:16048.

Kumar S, Suleski M, Craig JM, Kasprowicz AE, Sanderford M, Li M, Stecher G and Hedges SB (2022) TimeTree 5: An expanded resource for species divergence times. Mol Biol Evol 39:msac174.

Lassalle F, Spagnoletti M, Fumagalli M, Shaw L, Dyble M, Walker C, Thomas MG, Bamberg Migliano A and Balloux F (2018) Oral microbiomes from hunter-gatherers and traditional farmers reveal shifts in commensal balance and pathogen load linked to diet. Mol Ecol 27:182-195.

Lepp PW, Brinig MM, Ouverney CC, Palm K, Armitage GC and Relman DA (2004) Methanogenic archaea and human periodontal disease. Proc Natl Acad Sci U S A 101:6176-6181.

Li H (2011) A statistical framework for SNP calling, mutation discovery, association mapping and population genetical parameter estimation from sequencing data. Bioinformatics 27:2987-2993.

Liang G and Bushman FD (2021) The human virome: Assembly, composition and host interactions. Nat Rev Microbiol 19:514-527.

Mangola SM, Lund JR, Schnorr SL and Crittenden AN (2022) Ethical microbiome research with Indigenous communities. Nat Microbiol 7:749-756.

Matarazzo F, Ribeiro AC, Faveri M, Taddei C, Martinez MB and Mayer MP (2012) The domain archaea in human mucosal surfaces. Clin Microbiol Infect 18:834-840.

Minh BQ, Nguyen MAT and Von Haeseler A (2013) Ultrafast approximation for phylogenetic bootstrap. Mol Biol Evol, 30:1188-1195.

Mirzaei MK and Maurice CF (2017) Ménage à trois in the human gut: Interactions between host, bacteria and phages. Nat Rev Microbiol 15:397-408.

Möller ÅJ, Fabricius L, Dahlen G, Öhman AE and Heyden GUY (1981) Influence on periapical tissues of indigenous oral bacteria and necrotic pulp tissue in monkeys. Eur J Oral Sci 89:475-484.

Naud S, Ibrahim A, Valles C, Maatouk M, Bittar F, Tidjani Alou M and Raoult D (2022) Candidate phyla radiation, an underappreciated division of the human microbiome, and its impact on health and disease. Clin Microbiol Rev 6:e0014021.

Pride DT, Salzman J, Haynes M, Rohwer F, Davis-Long C, White III RA, Loomer P, Armitage GC and Relman DA (2012) Evidence of a robust resident bacteriophage population revealed through analysis of the human salivary virome. ISME J 6:915-926.

Puzio N, Sikora M, Srebrna A, Straczek A, Weglarz N, Lewandowska K, Mazurek G and Thum-Tyzo K (2021) Symptoms of selected parasitic diseases in the oral cavity. J Pre-Clin Clin Res 15:34-39.

Rozas J, Ferrer-Mata A, Sánchez-DelBarrio JC, Guirao-Rico S, Librado P, Ramos-Onsins SE and Sánchez-Gracia A (2017) DnaSP 6: DNA sequence polymorphism analysis of large data sets. Mol Biol Evol 34:3299-3302.

Salzburger W, Ewing GB and Von Haeseler A (2011) The performance of phylogenetic algorithms in estimating haplotype genealogies with migration. Mol Ecol 20:1952-1963.

Sela I, Ashkenazy H, Katoh K and Pupko T (2015) GUIDANCE2: Accurate detection of unreliable alignment regions accounting for the uncertainty of multiple parameters. Nucleic Acids Res 43:W7-W14.

Shkoporov AN, Khokhlova EV, Fitzgerald CB, Stockdale SR, Draper LA, Ross RP and Hill C (2018) ΦCrAss001 represents the most abundant bacteriophage family in the human gut and infects Bacteroides intestinalis. Nat Commun 9:4781.

Socransky SS, Haffajee AD, Cugini MA, Smith CKJR and Kent Jr RL (1998) Microbial complexes in subgingival plaque. J Clin Periodontol 25:134-144.

Szafrański SP, Kilian M, Yang I, Bei der Wieden G, Winkel A, Hegermann J and Stiesch M (2019) Diversity patterns of bacteriophages infecting *Aggregatibacter* and *Haemophilus* species across clades and niches. ISME J 13:2500-2522.

Tan Y and Li C (2021) Protozoa and oral health: A systematic review. J Biol Regul Homeost Agents 35:47-54.

Trifinopoulos J, Nguyen LT, von Haeseler A and Minh BQ (2016) W-IQ-TREE: A fast online phylogenetic tool for maximum likelihood analysis. Nucleic Acids Res 44:W232–W235.

Uzel NG, Teles FR, Teles RP, Song XQ, Torresyap G, Socransky SS and Haffajee AD (2011) Microbial shifts during dental biofilm re-development in the absence of oral hygiene in periodontal health and disease. J Clin Periodontol 38:612-620.

Van de Peer Y (1996) A quantitative map of nucleotide substitution rates in bacterial rRNA. Nucleic Acids Res 24:3381-3391.

Woese C (1987) Bacterial evolution. Microbiol Rev 51:221-271.

Internet Resources

Human Oral Microbiota Database (HOMD) (2023) Human Oral Microbiota Database v. 3.1, https://www.homd.org/ (accessed 8 and 14 February 2022; accessed April 12th and 19th 2022).

R Core Team (2021) R: A language and environment for statistical computing, R Foundation for Statistical Computing, Vienna, Austria, https://www.R-project.org/ (accessed April 26, 2023).

Rambaut A (2018) FigTree v.1.4.4, http://tree.bio.ed.ac.uk/software/figtree/ (accessed April 23, 2023).
